# Sensor System Design and Deployment for Environmental and Visitor Monitoring in Cultural Heritage Sites

**DOI:** 10.3390/s26154941

**Published:** 2026-08-04

**Authors:** Sofía Aparicio, Fernando Ramonet, Lidia Abad, Darío Sánchez, Antonio de la Cruz, Tianyu Yin, Rundong Zhang, José Javier Anaya

**Affiliations:** 1 Institute of Physical and Information Technologies Leonardo Torres Quevedo (ITEFI), Spanish National Research Council (CSIC), C/Serrano 144, 28006 Madrid, Spain; fernando.ramonet@csic.es (F.R.); lidia.abad@csic.es (L.A.); dario.sanchez@csic.es (D.S.); antonio.cruz@csic.es (A.d.l.C.); tianyu.yin@alumnos.upm.es (T.Y.); rundong.zhang@alumnos.upm.es (R.Z.); jj.anaya@csic.es (J.J.A.); 2 Escuela Técnica Superior de Ingenieros Industriales, Universidad Politécnica de Madrid, C. José Gutiérrez Abascal 2, 28006 Madrid, Spain

**Keywords:** WSNs, monitoring, IoT sensors, cultural heritage

## Abstract

In this paper the design, development, and validation of a set of low-power low-cost sensor systems intended for environmental monitoring and visitor tracking in cultural heritage sites are shown. These systems have been deployed in five pilot cases in four different countries in Europe inside the European ARGUS project. Two different approaches have been defined in the design: the static systems installed in fixed positions and the dynamic systems installed in a moving robot, i.e., a quadruped robot or a drone. The Baltanás pilot site has served as the primary testing platform, enabling accelerated prototyping and iterative improvement before deployment across additional pilot locations. This paper presents the design criteria, system architectures, and performance evaluation of these sensor networks, including thermal–humidity probes, volumetric water content sensors, wind measurement systems, pollution monitors, and two generations of visitor counting devices.

## 1. Introduction

The preservation of cultural heritage (CH) is essential for safeguarding the historical, cultural, and social identity of societies. Archaeological sites, historic buildings, museums, monuments, and underground heritage are continuously exposed to environmental factors, natural hazards, human activities, and aging processes that contribute to their gradual deterioration. Preventive conservation strategies increasingly rely on continuous monitoring to detect early signs of degradation, enabling timely interventions while minimizing restoration costs and avoiding irreversible damage. Consequently, the development of reliable, non-invasive, low-cost and scalable monitoring technologies has become one of the main research priorities in CH conservation.

Wireless sensor networks (WSNs) and Internet of Things (IoT) technologies have significantly expanded the possibilities for long-term monitoring of CH. Compared with conventional wired systems, wireless solutions can be deployed with minimal intervention on historically sensitive structures, reducing installation costs, maintenance requirements, and visual impact while providing continuous environmental and structural information.

Although numerous monitoring solutions have been proposed in recent years, most address specific conservation problems or individual sensing modalities. Comprehensive frameworks integrating heterogeneous sensing technologies, visitor monitoring, and autonomous robotic inspection remain scarce, particularly when validated across multiple heritage environments. Furthermore, scalable low-cost architectures capable of supporting preventive conservation in diverse CH scenarios are still limited.

To address these challenges, the European ARGUS project (Non-destructive, scalable, smart monitoring of remote cultural treasures) [1] develops an integrated technological framework for preventive conservation based on distributed sensing, autonomous robotic platforms, cloud services, and intelligent data analysis. This work presents the wireless sensing technologies developed within ARGUS for environmental monitoring and visitor tracking.

The aim of this work is to design, implement, and experimentally validate a family of low-cost low-power wireless sensing systems for environmental monitoring and visitor counting in CH environments. The proposed solutions include fixed wireless sensor nodes together with mobile sensing platforms integrated into autonomous robotic systems, enabling complementary monitoring strategies adapted to different conservation scenarios. The designed systems were initially deployed, tested, optimized and validated in the underground wine cellars of Baltanás (Spain) before being deployed and validated in five pilot heritage sites distributed across four European countries.

The main contributions of this work are:

- The design of a modular and scalable wireless monitoring architecture specifically tailored for CH applications;
- The development of a family of low-cost low-power sensing systems for environmental monitoring and visitor counting;
- The integration of fixed sensing nodes with mobile sensing platforms mounted on autonomous robotic systems, including an UAV and a quadruped robot;
- The validation of the proposed technologies through real deployments in five heterogeneous European CH sites;
- The experimental evaluation of the proposed systems in terms of functionality, reliability, scalability, and suitability for preventive conservation in different conditions.

The remainder of this paper is organized as follows. Section 2 reviews the state of the art in wireless monitoring technologies for CH and briefly introduces the ARGUS project. Section 3 describes the monitoring systems developed by the authors. Section 4 discusses the experimental validation and results. Finally, Section 5 summarizes the main conclusions of this work.

## 2. State of the Art and Project Context

### 2.1. State of the Art of Wireless Monitoring in Cultural Heritage

Early research on wireless monitoring for CH primarily focused on environmental and structural health monitoring using WSNs. These pioneering studies demonstrated the feasibility of WSNs as a non-invasive alternative to conventional wired monitoring systems, enabling continuous acquisition of parameters such as temperature, relative humidity, vibrations, crack evolution, rainfall, and structural deformation while preserving the integrity of heritage assets [2,3,4,5,6]. Subsequent research expanded the scope of wireless sensing to indoor environmental quality assessment, preventive conservation of museum collections, and large-scale monitoring through distributed sensing platforms [6,7,8]. Recent systematic reviews and bibliometric studies identify wireless sensing as one of the key enabling technologies for smart heritage preservation, supporting long-term conservation through continuous monitoring and early deterioration detection [9,10,11].

The evolution of WSNs has progressively led to the adoption of Internet of Things (IoT) architectures, which enhance monitoring capabilities by providing remote access, cloud-based data management, interoperability, and scalable system deployment [8,9,12,13,14,15,16]. More recently, intelligent monitoring approaches have emerged through the integration of artificial intelligence (AI), digital twins, and predictive maintenance techniques, enabling monitoring systems not only to collect data but also to detect deterioration patterns, estimate degradation risks, and support conservation decision making [9,13,17,18,19,20,21]. In particular, AI-assisted visual inspection, machine learning algorithms, and digital twin technologies are increasingly employed to automate damage assessment, optimize maintenance planning, and improve the management of heritage assets [10,11,17,18,19,20,21,22]. Recent bibliometric analyses further highlight IoT, AI, digital twins, remote sensing, and cyber-physical systems as the main technological drivers of next-generation smart heritage monitoring platforms [10,11,17].

Despite these significant advances, several challenges remain. Most existing solutions are designed for a single monitoring objective, such as environmental sensing or structural health assessment, while integrated architectures combining heterogeneous sensing modalities are still limited. Likewise, only a limited number of studies have combined fixed WSNs, IoT connectivity, visitor monitoring, and autonomous robotic inspection within a unified monitoring framework, and even fewer have validated their approaches across multiple heterogeneous heritage sites [13,17,18,23,24]. These limitations, together with the lack of low-cost and open monitoring solutions that can be easily integrated with existing heritage management infrastructures, motivate the development of the integrated wireless monitoring architecture proposed in this work. The proposed architecture is based on a set of modular monitoring systems designed to provide scalability, interoperability, and adaptability across different cultural heritage scenarios.

### 2.2. The ARGUS Project

ARGUS (Non-destructive, scalable, smart monitoring of remote cultural treasures) [1] is a Horizon Europe research project aimed at developing an integrated framework for the preventive conservation of CH. The project combines wireless sensing, autonomous robotic inspection, digital twins, AI-based data analysis, and cloud technologies to support continuous monitoring and informed conservation decision making.

The technologies are being validated in five pilot sites representing different heritage typologies and conservation challenges: the archaeological site of Delos (Greece), Schenkenberg Castle (Switzerland), the underground wine cellars of Baltanás (Spain), Monti Lucretili (Italy), and the Abbey of Sant’Antonio di Ranverso (Italy). These sites provide heterogeneous environmental and operational conditions for evaluating the scalability and adaptability of the proposed monitoring solutions.

The present paper focuses on the design, implementation, and validation of the wireless sensing systems developed within ARGUS for environmental monitoring and visitor tracking. These systems constitute one of the core technological components of the overall ARGUS framework and provide the experimental basis for the validation presented in the following sections.

## 3. Materials and Methods

In this section the monitoring systems developed by the authors are introduced.

Most commercial monitoring systems used in structural and environmental monitoring of CH are generally based on proprietary architectures that limit interoperability and the integration of application-specific sensors, which is a critical requirement in heterogeneous heritage environments. Recent reviews highlight that traditional structural health monitoring (SHM) solutions are often fragmented and rely on closed or semi-closed systems, which reduces their adaptability and long-term sustainability in CH applications [25]. In addition, these systems are frequently associated with high deployment and maintenance costs, as well as scalability limitations when applied to large or complex heritage sites [9]. From an operational perspective, conventional monitoring infrastructures may also require significant energy and maintenance resources, which restricts their suitability for continuous long-term deployments in fragile or remote environments [26]. Consequently, recent research has increasingly focused on WSNs and IoT-based architectures that enable low-cost, energy-efficient, and modular monitoring systems, improving flexibility and enabling the integration of heterogeneous sensing technologies tailored to CH conservation needs [15].

To overcome these limitations, the authors designed custom LoRaWAN-based systems using commercially available electronics and sensors. These systems are tailored to the particular needs of this study, allowing for flexible sensor integration and low-power operation.

**Figure 1:**
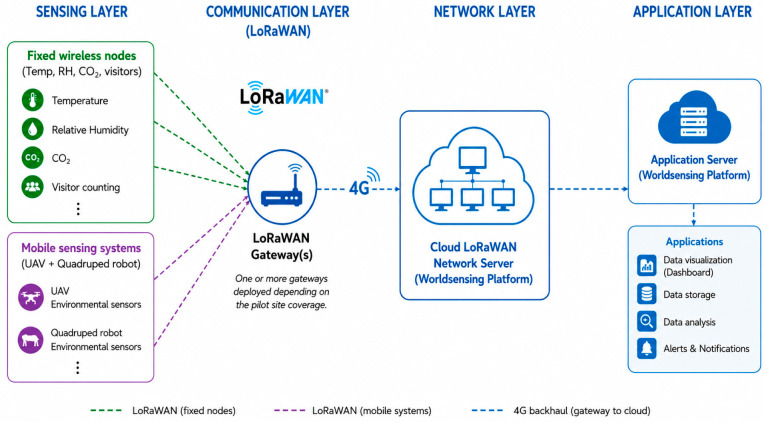
Overall architecture of the LoRaWAN-based monitoring approach developed in the ARGUS project, including fixed wireless sensing nodes, mobile sensing platforms, LoRaWAN communication infrastructure, cloud services, and user applications.

Figure 1 presents the overall architecture of the proposed monitoring framework. The system is based on a long-range wide area network (LoRaWAN) communication infrastructure that integrates both fixed wireless sensing nodes and mobile sensing platforms into a common architecture. Environmental and operational data collected by the sensing devices are transmitted through LoRaWAN gateways to the network server and subsequently to the application server, where they are stored, visualized, and analyzed.

During the development and experimental validation stages, the ThingSpeak platform (MathWorks, Natick, MA, USA) was used because it provides a simple, reliable, and easy-to-configure cloud environment for rapid prototyping, real-time data visualization, and system verification. Its use facilitated the testing of the sensing nodes and the communication infrastructure before their integration into the complete ARGUS framework.

The final operational deployment relies on the proprietary cloud platform developed by Worldsensing [27], one of the project partners. Depending on the monitoring area and the required radio coverage, one or more LoRaWAN gateways were deployed at each pilot site. The number and placement of the gateways were adapted to the specific characteristics and communication requirements of each deployment. For example, in the Baltanás underground wine cellar pilot, radio frequency signal attenuation caused by the underground environment significantly reduced the communication range of the LoRaWAN nodes. To ensure reliable data transmission from sensors installed in the deepest sections of the cellar, an additional LoRaWAN gateway was installed inside the underground facility. This example illustrates one of the main deployment challenges encountered during the project and how the communication infrastructure was adapted to guarantee reliable network coverage. The gateways received the data transmitted by the sensing nodes and forwarded the LoRaWAN packets to the Worldsensing cloud platform through a 4G mobile backhaul connection. The cloud platform integrates the LoRaWAN network server functionality with the ARGUS data management platform, enabling centralized data storage, visualization, and analysis across all pilot sites. Owing to the commercial nature of this infrastructure, its internal implementation details are beyond the scope of this work.

Several static and dynamic monitoring systems have been designed, developed and tested for the ARGUS project. Components were selected based on five criteria: (i) small physical footprint, (ii) low power consumption, (iii) low manufacturing cost, (iv) LoRaWAN compatibility, and (v) type-specific performance specifications. Table 1 summarizes the wireless sensing systems developed within the ARGUS project. These systems constitute the sensing layer of the proposed monitoring architecture and are deployed according to the specific characteristics of each CH pilot site. Static systems are based on fixed LoRaWAN wireless nodes for continuous environmental and visitor monitoring, whereas dynamic systems are integrated into autonomous robotic platforms to perform mobile inspections in areas that are difficult to access using fixed infrastructure. These systems were based on commercial Arduino MKR WAN 1310 platforms, which provide native LoRaWAN connectivity through an integrated Semtech SX1276 LoRa transceiver, enabling long-range low-power wireless communication.

### 3.1. Static Systems

Static monitoring systems in CH environments refer to fixed or permanently installed sensing infrastructures designed to continuously collect environmental and usage-related data without mobility. These systems typically focus on long-term in situ measurements of key parameters relevant to conservation and structural assessment. As summarized in the table, static deployments include movement detection and airflow monitoring, visitor and people counting systems (including both basic and enhanced visitor monitoring approaches), as well as environmental sensing such as internal air temperature and relative humidity measurements at different depths within the structure. They also encompass more specialized measurements such as volumetric water content (VWC) combined with internal thermal–hygrometric conditions, wind direction and velocity monitoring, and pollutant concentration assessment. In contrast to dynamic systems, which rely on mobile platforms such as unmanned aerial vehicles (UAVs) or quadruped robots for gas sensing in specific scenarios, static systems provide continuous, distributed, and long-term observations that are essential for understanding gradual environmental changes and their impact on CH assets.

#### 3.1.1. Indoor Movement and Airflow Monitoring System

To measure both human entry and ventilation conditions in this case study, an integrated wireless monitoring system was developed. The system combines a PIR HC-SR501 sensor (Utmel Electronic Ltd., Hong Kong, China) for visitor detection and an Omron DF6V Micro-Electro-Mechanical Systems (MEMS) airflow sensor (Omron Corporation, Kyoto, Japan), both integrated with an Arduino MKR WAN 1310 board (Arduino S.r.l., Ivrea, Italy), which provides LoRaWAN connectivity for long-range low-power wireless communication.

**Figure 2:**
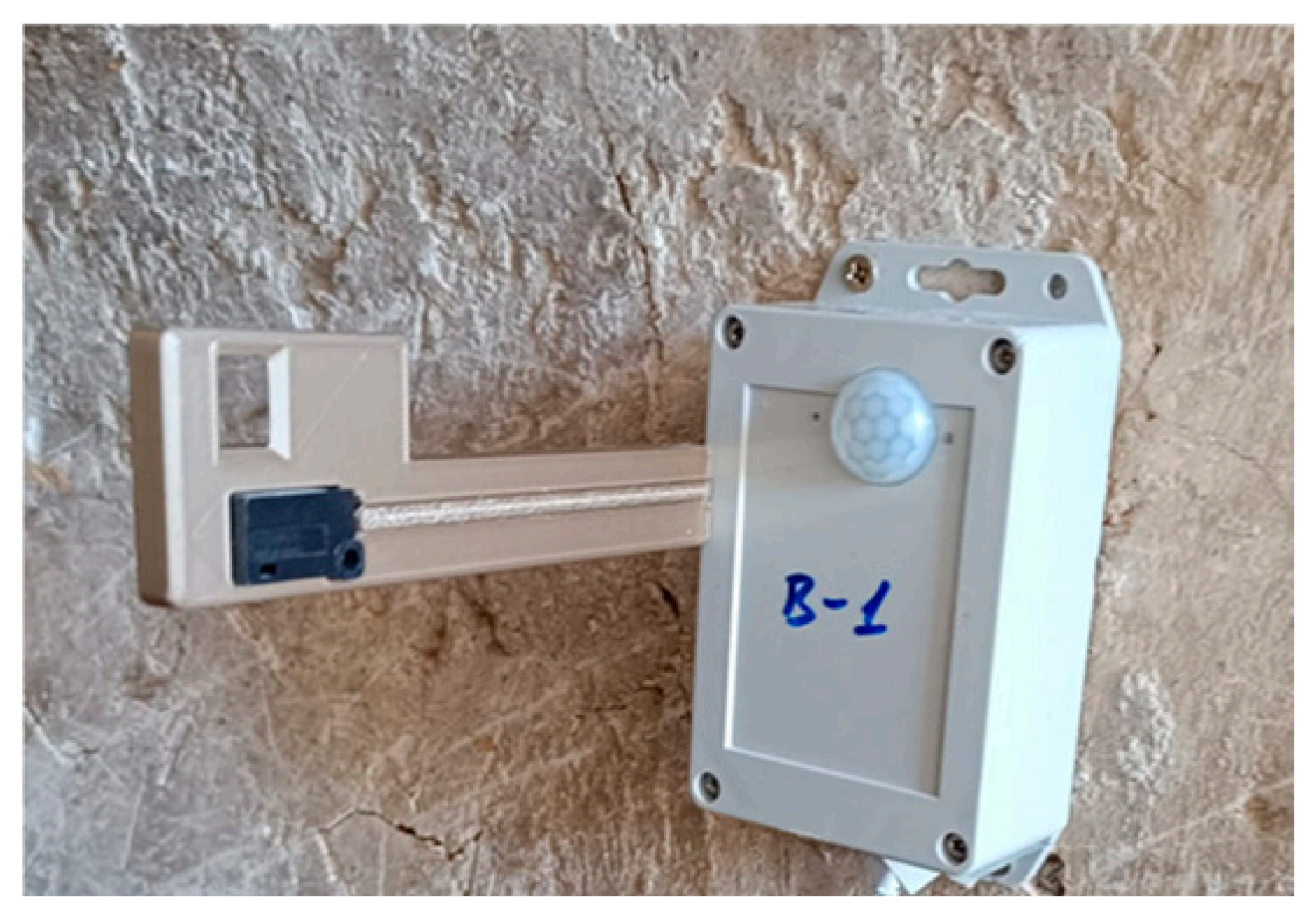
Wireless LoRaWAN sensing node developed for visitor detection and airflow monitoring.

Figure 2 illustrates the developed sensing node. The electronic components are housed inside a commercial enclosure to protect them from the harsh environmental conditions typically found in underground heritage sites. The PIR sensor is mounted on the front face of the enclosure to detect visitor movement, while the MEMS airflow sensor is installed on a custom 3D-printed support that positions the sensing element outside the enclosure. This configuration minimizes airflow disturbances caused by the enclosure and improves the accuracy of airflow measurements near the cellar entrance. The system was deployed at the entrances of two underground wine cellars in Baltanás, where it simultaneously monitored visitor access and natural ventilation conditions, both of which are closely related to the high humidity levels affecting the conservation of the site. To correlate airflow patterns with the opening and closing of the cellar door, the sensors were installed directly at the entrance, enabling the relationship between door operation and natural ventilation to be accurately assessed. These historic cellars incorporate multiple ventilation openings, originally designed to regulate the internal microclimate during the wine production process. As natural airflow strongly influences the temperature and relative humidity inside the cellars, monitoring air circulation is essential for understanding the ventilation dynamics and assessing their impact on the preservation of the underground heritage environment.

#### 3.1.2. Outdoor Visitor and Vehicle Monitoring System

The main objective of the visitor monitoring subsystem is to accurately count the number of people or objects passing through specific channels (such as entrances, exits, or corridors) and to precisely determine their direction of movement. Its hardware composition is relatively simple, consisting primarily of a central controller and an infrared sensor unit.

Most of the systems for that purpose are based on cameras and their consumption is very high or they are prepared for indoors.

Two different versions have been developed. From a very simple proof-of-concept prototype (V1), its limitations were analyzed, and it subsequently evolved into an improved version (V2) with enhanced functionality and a more comprehensive decision-making logic.

Prototype V1 consists of an Arduino MKR WAN 1310, an infrared photoelectric sensor (SICK VL180-2N41131, SICK AG, Waldkirch, Germany) and a reflector, and a LoRa antenna. The externally powered sensor detects the passage of objects by identifying interruptions in a reflected infrared beam, and the system attempts to classify pedestrians and vehicles based on the duration of the beam obstruction. Its operating principle is as follows: the emitter continuously transmits an infrared light beam, and the reflector installed on the opposite side accurately reflects the beam back to the receiver, which is integrated within the same sensor module. When an opaque object (such as a pedestrian or a vehicle) passes through and blocks the beam, the receiver no longer detects the reflected light, and the digital output signal changes state.

The software performs a preliminary classification of passing objects by measuring the total duration of the infrared beam interruption. For example, based on experimental data, a threshold is defined whereby events with a blockage duration of less than 1.5 s are classified as “pedestrians”, those between 1.5 and 6 s as “vehicles”, and those exceeding 10 s as abnormal occupancies, which trigger an alarm.

However, this first version presents several limitations. It cannot determine the direction of movement, as it only registers that an object has passed, and its accuracy is limited because the classification method—relying solely on the time the beam is blocked—is unreliable in real-world scenarios.

These shortcomings motivated the development of a more advanced V2 prototype. To evaluate the performance of V1, the system was tested for approximately one week in a 1.5 m wide corridor at the Institute of Physical and Information Technologies (ITEFI) of CSIC, with the sensor and reflector mounted on opposite sides at a height of 1 m.

To overcome the key functional limitations of Version V1, the V2 prototype underwent a significant hardware upgrade, most notably through the addition of a second reflective photoelectric sensor. The two sensors are placed side by side along the walking direction, separated by a small fixed distance (typically 10–20 cm). The main components of the system include an Arduino MKR WAN 1310, two reflective photoelectric sensor modules (the same model as in the first version with their corresponding reflectors), and a LoRa antenna. Because the physical appearance of these components was already shown in the V1 description, it is not repeated here.

Although the hardware modification appears simple, it provides the logical foundation required for determining direction. The principle is similar to industrial quadrature encoders: when an object passes, it activates the two sensors in sequence, and, by analyzing which sensor is triggered first, the firmware can accurately determine the direction of movement. If sensor A is blocked first and sensor B second, the system registers an entry; if sensor B is triggered before sensor A, it records an exit.

To fundamentally address the aforementioned issues, firmware version V2 restructures the core logic using a finite state machine (FSM) approach. The FSM abstracts the system into a finite number of states and clearly defines state transitions and the actions to be executed when a specific event is triggered. In embedded systems, the FSM methodology enables complex and intertwined logic to be divided into maintainable and predictable units. Moreover, it compels developers to systematically consider all possible states and transitions prior to implementation, thereby reducing logical vulnerabilities.

In addition, the firmware incorporates an anti-bounce delay of approximately 20 ms when processing sensor inputs. A state change is only confirmed if it remains stable for this duration, effectively reducing misinterpretations caused by mechanical vibrations.

Compared with a simple if-then-else logic structure, this FSM-based design provides a clearer architecture and more rigorous logical framework. It effectively prevents misclassification caused by signal fluctuations or complex traffic situations (such as direction changes during passage). This approach represents a method for achieving reliable direction determination.

This enhancement transforms the system from merely counting objects to offering directional counting, greatly increasing its practical value.

To investigate the relationship between vehicle traffic and potential vibration events inside the cellars, Version 1 of the system was installed outside two cellars in Baltanás. Since the direction of vehicle movement was not required for this analysis, the system was configured solely to detect passing vehicles and correlate their occurrence with vibration measurements collected within the cellars.

#### 3.1.3. System to Measure Internal Ambient TH at Different Depths

**Figure 3:**
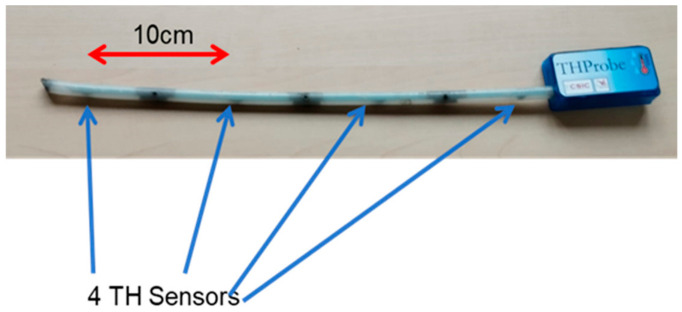
Sensor developed to measure internal TH.

A system to monitor internal ambient TH was designed by the authors, see Figure 3. The sensors measure the conditions inside the material’s chamber using an Arduino-based platform. They are digital sensors with adaptable probe lengths. Four temperature and humidity (TH) sensors are integrated into each device to simultaneously monitor the microclimatic conditions at different locations inside the monitored material or chamber. This configuration enables the identification of spatial TH gradients, which are particularly relevant in CH applications where localized environmental variations can significantly influence material deterioration. The TH sensors employed are commercial Honeywell HDC302x-Q1 (Honeywell, Charlotte, NC, USA) devices, characterized by very low power consumption (<1 mA). The system operates on a 3.7 V, 2.6 mAh battery.

A first version of this prototype was tested in Schenkenberg Castle.

#### 3.1.4. System to Measure VWC and Internal Ambient TH

**Figure 4:**
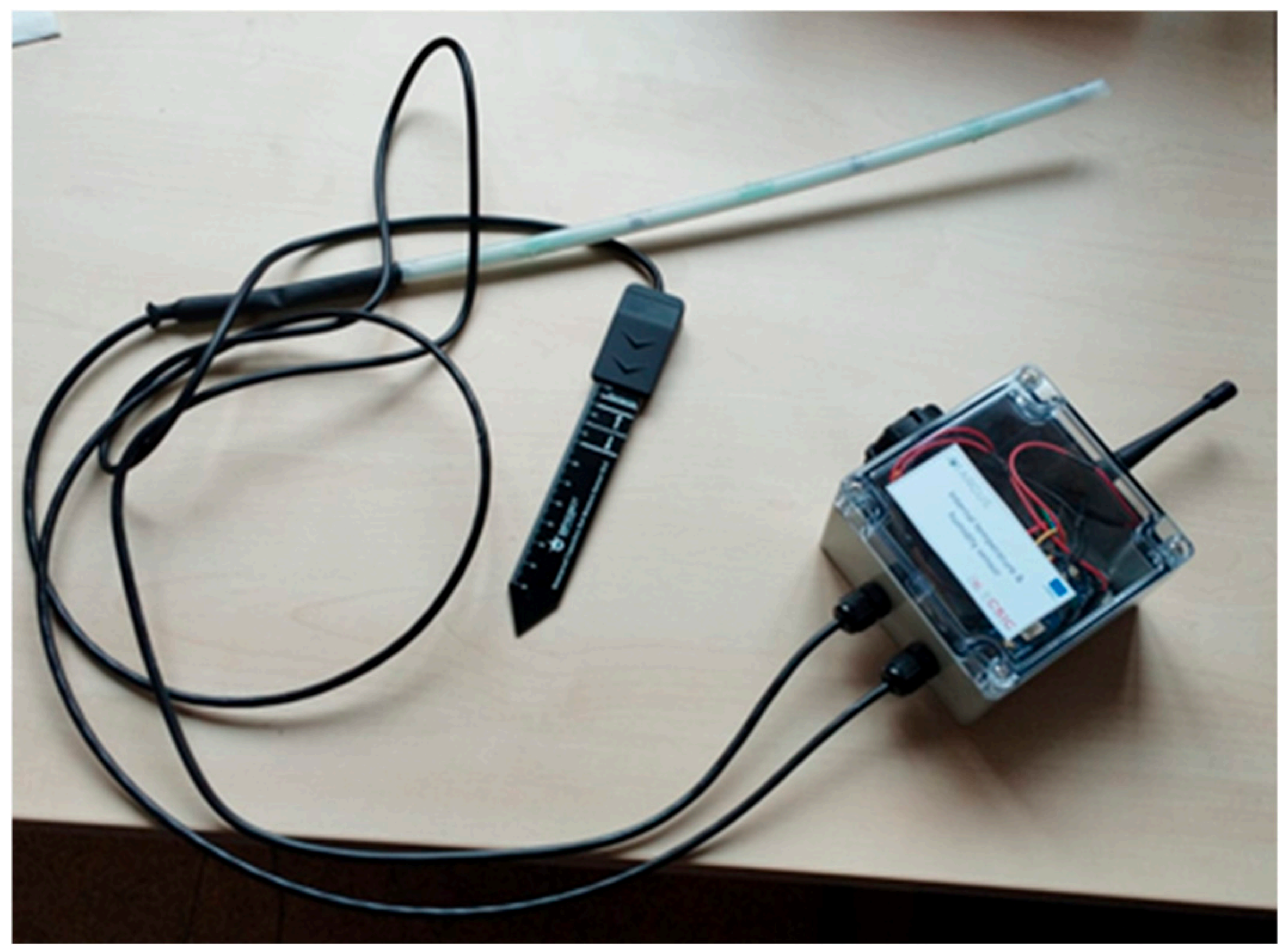
Custom-built system developed to measure internal wall temperature, relative humidity, and VWC.

A custom-built system was developed to measure internal wall temperature, relative humidity, and VWC, see Figure 4. The system is based on an Arduino MKR WAN 1310 and integrates the previously developed TH sensor together with the commercial SEN0193 (DFRobot, Shanghai, China) to determine VWC. The SEN0193 is an analog capacitive sensor characterized by very low power consumption. Although the SEN0193 sensor was originally developed for soil moisture measurements, it operates by measuring variations in the dielectric permittivity of the surrounding medium. Since the dielectric properties of porous building materials are also strongly influenced by their water content, dielectric sensing techniques have been widely investigated for moisture monitoring in masonry and cement-based materials [28]. Therefore, in this work the sensor was used as a low-cost device to monitor relative temporal variations in moisture within wall cracks rather than to determine the absolute moisture content of the construction material. In the proposed system, the sensor probes are inserted into selected points of the wall or crack, enabling continuous monitoring of internal moisture variations associated with water infiltration and material deterioration. The system is powered by a 3.6 V, 2.6 Ah AA battery.

Two systems were deployed at the Abbey of Sant’Antonio di Ranverso to monitor temperature, relative humidity, and moisture inside two representative wall cracks, while an additional system was installed at Schenkenberg Castle to evaluate the applicability of the proposed approach under different heritage conservation conditions.

#### 3.1.5. Wind Speed and Direction Measurement Prototypes

Two prototype systems for measuring wind speed and direction have been developed using MEMS airflow sensors. The first prototype (V1) is designed for low wind speeds up to 10 km/h, while the second prototype (V2) extends the measurement range to 54 km/h. Both systems share a similar architecture based on a low-power microcontroller and multiple airflow sensors arranged in different orientations to estimate wind direction.

V1: Low-speed prototype (0–10 km/h).

**Figure 5:**
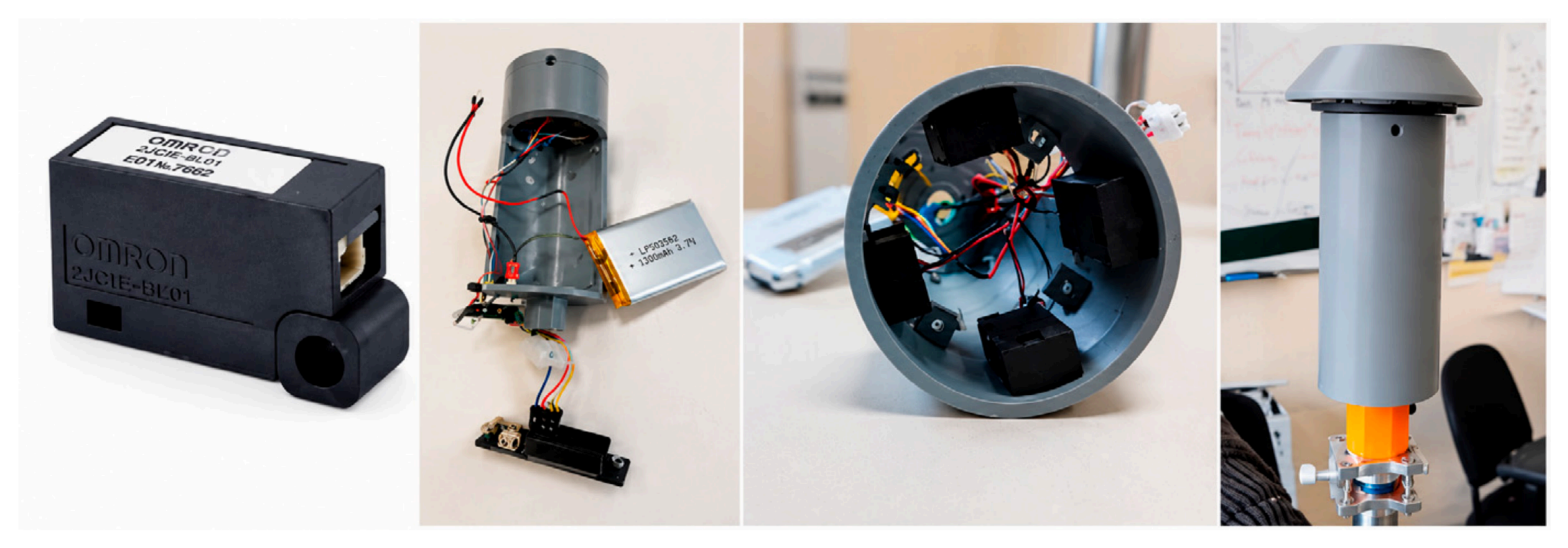
Prototype V1 of 4-direction anemometer, velocities up to 10 km/h.

This system is based on an Arduino MKR WAN 1310 microcontroller and incorporates four Omron D6F-V03A1 airflow sensors, see Figure 5. Each sensor is oriented toward one of the cardinal directions (north, south, east, and west), enabling the estimation of both wind speed magnitude and direction. These MEMS airflow sensors are capable of measuring air velocities up to 10 km/h, making the system suitable for environments with low air movement.

V2: Extended-range prototype (0–54 km/h).

**Figure 6:**
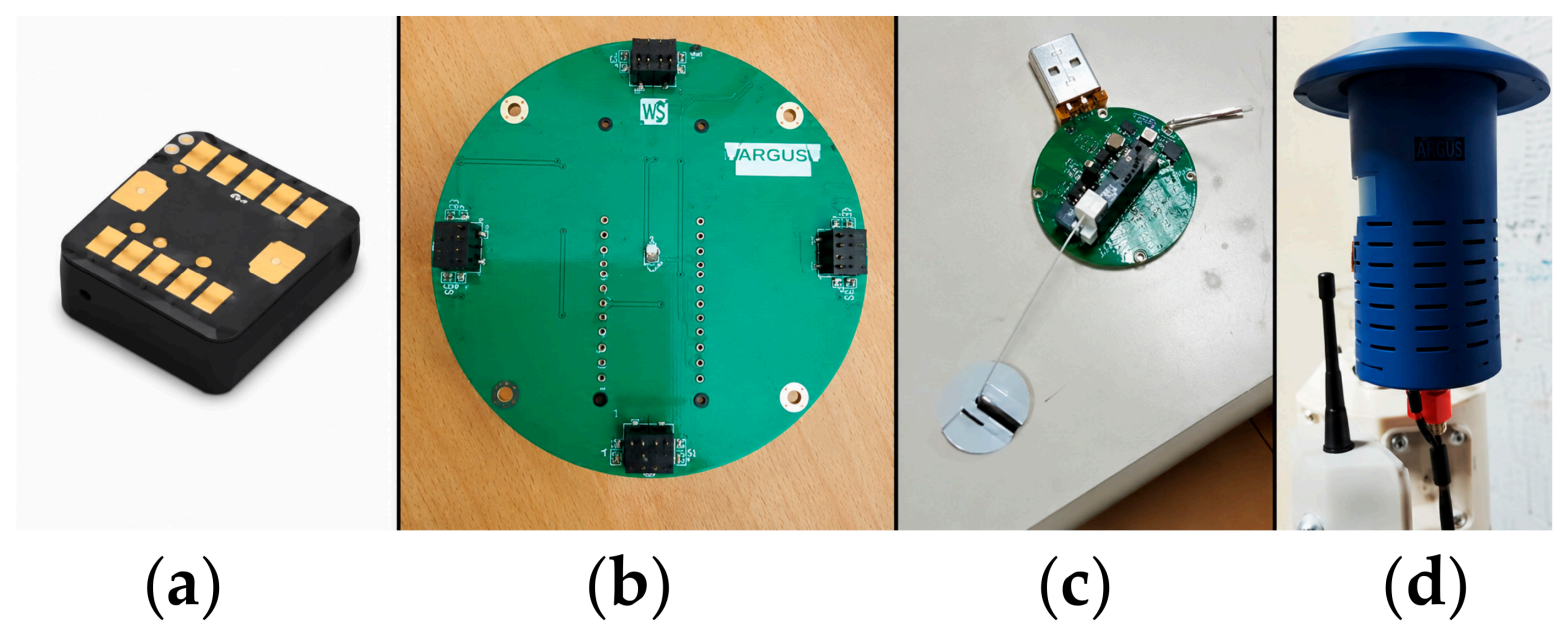
(**a**) FS3000-1015 air velocity sensor extracted from sparkfun.com, (**b**) electronic board with 4 air velocity sensors, (**c**) electronic board with the Arduino and the battery, and (**d**) first encapsulated prototype V2 developed with a 3D printer.

The second prototype also uses an Arduino MKR WAN 1310 microcontroller but employs four Renesas (FS3000-1015, Renesas Electronics, Tokyo, Japan) airflow sensors, see Figure 6. These sensors are based on an MEMS thermopile sensing element and provide digital output with 12-bit resolution, allowing wind speed measurements of up to 54 km/h. As in the first prototype, the four sensors are arranged in different orientations to estimate wind direction.

In addition to airflow sensing, the system incorporates an (SHT35, Sensirion AG, Stäfa, Switzerland) sensor to measure TH with high accuracy, providing environmental context for the wind measurements.

Both prototypes are designed for low-power operation, enabling long-term autonomous deployments powered by batteries. The Arduino MKR WAN 1310 provides integrated LoRaWAN communication, allowing the collected data—wind speed, wind direction, temperature, and humidity—to be transmitted wirelessly over long distances. This design makes the system suitable for environmental monitoring, field experiments, and scientific studies, particularly in remote locations where power availability is limited.

A new version of the system was developed and equipped with a solar panel to allow it to operate without external power.

Two V2 systems were deployed in two case studies: one in Delos and another at Ranverso Abbey.

#### 3.1.6. System to Measure Pollution

The authors have been testing various low-cost low-power sensors to measure different gases, including CO_2_, CO, NO_2_, NH_3_, SO_2_, particulate matter (PM), and volatile organic compounds (VOCs). The sensors selected for the prototype are shown in Table 2.

**Figure 7:**
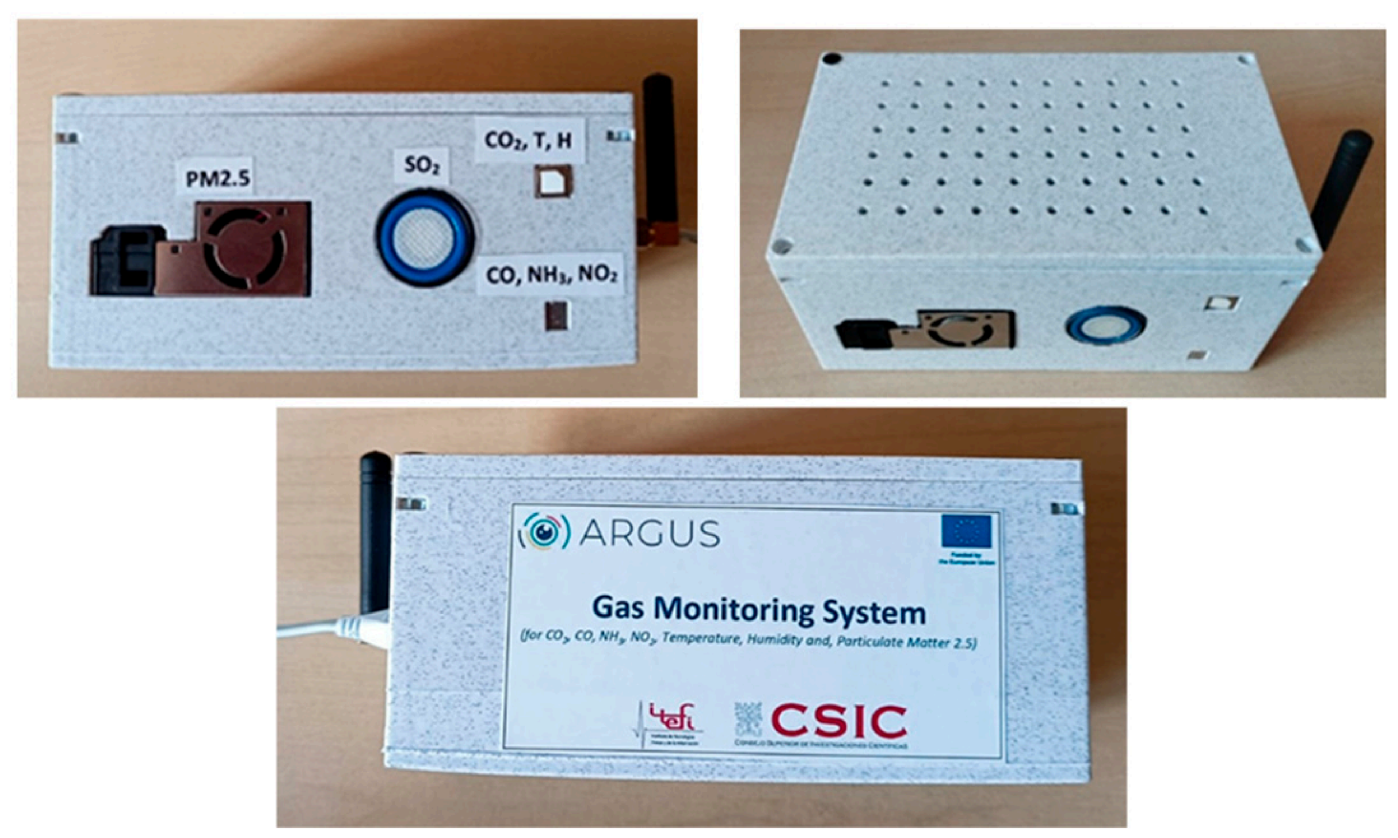
Final version of the gas system for indoors.

A custom enclosure was designed using Autodesk Inventor professional 2015 (Autodesk, San Francisco, CA, USA).software. Manufacturing was carried out through additive manufacturing (3D printing). The three-dimensional design was developed considered essential criteria of functionality, protection, and non-invasive deployment. The final version of the prototype is shown in Figure 7.

### 3.2. Dynamic Systems

Dynamic monitoring systems in CH refer to approaches where the sensing and data acquisition tasks are performed by mobile platforms rather than fixed installations. Unlike static systems, these solutions rely on movable devices such as UAVs, ground robots, or portable sensing units that can be deployed on demand to inspect specific areas of interest. This mobility enables flexible, high-resolution, and targeted monitoring of inaccessible or hazardous zones, such as upper facades, roofs, or structurally complex regions. Typical applications include gas and pollutant detection, visual inspection, and environmental sampling in localized areas where static sensor deployment is not feasible or sufficient.

The use of mobile platforms also enables rapid inspections following exceptional events, such as heavy rainfall, flooding, fires, earthquakes, landslides, or structural damage, allowing additional measurements to be acquired only in the areas where anomalies have been detected by the fixed monitoring infrastructure. UAVs are particularly suitable for inspecting elevated structures and large outdoor areas, whereas quadruped robots can operate in confined or hazardous environments where aerial inspection is impractical, such as underground wine cellars, tunnels, galleries, or areas with uneven terrain. Equipped with environmental sensors and imaging devices, quadruped robots can safely access these locations while reducing the need for personnel to enter potentially hazardous environments.

However, dynamic systems are generally used for short-term or episodic measurements due to constraints in battery life, operational complexity, and limited continuous coverage. As a result, they are often considered complementary to static monitoring infrastructures, providing additional spatial detail and on-demand inspection capabilities within integrated CH monitoring strategies.

Among the gas monitoring systems developed in the project, several configurations have been designed and tested. All the systems described so far are intended for static monitoring, meaning they are installed in a fixed location. To complement the fixed monitoring network, two additional systems based on a UAV and a quadruped robot were developed for dynamic monitoring. These platforms can be deployed periodically for preventive inspections or on demand when the fixed sensor network identifies abnormal environmental conditions requiring further investigation. To monitor large areas, hard-to-reach locations, or sporadically relevant zones, two additional systems were created for dynamic monitoring.

#### 3.2.1. UAV-Mounted Ultralight System

An ultralight system was designed to be placed on a UAV. The system was composed of a light microcontroller, the Adafruit Feather M0 WiFi—ATSAMD21 board (Adafruit Industries, Brooklyn, NY, USA). This microcontroller was chosen for its light weight, only 6.1 g. The lightweight system (under 130 g including the battery) designed by the authors is available in two different chimney configurations, vertical and horizontal. Both configurations include the same set of gas sensors, (MiCS-6814, Amphenol SGX Sensortech, Corcelles-Cormondrèche, Switzerland) sensor for CO, NH_3_ and NO_2_ and Sensirion SCD41 (Sensirion AG, Stäfa, Switzerland) for CO_2_, temperature, and humidity.

A measurement was performed every 10 s and was sent using WiFi to the cloud.

**Figure 8:**
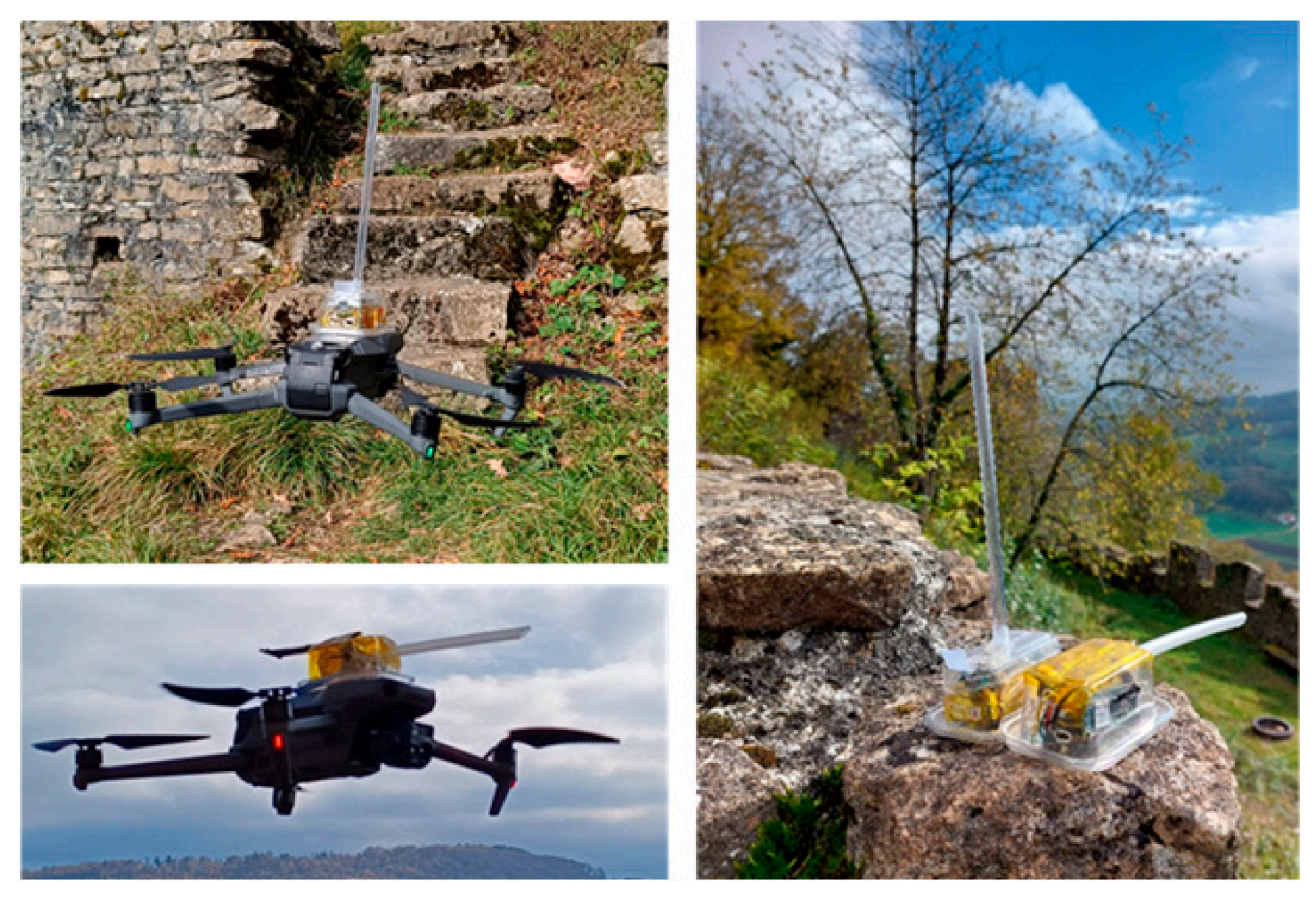
UAV with two chimney configurations.

The sensors are housed inside the drone’s mounting box, which incorporates a vertical chimney-like ventilation structure. In one configuration, the chimney faces forward; in the other, it faces upward. Figure 8 shows both systems. This system was tested on the Pilot Site of Schenkenberg in Switzerland. The sensing unit is mounted directly on the UAV, allowing environmental measurements to be acquired during flight over different areas of the heritage site. This mobile configuration enables rapid inspection of locations that are difficult to access with fixed sensor nodes while providing spatially distributed environmental information without requiring permanent sensor deployment. The first results are presented in [29].

#### 3.2.2. Quadruped Robot System

The proposed system uses the Unitree GO2 quadruped robot as its mobile platform, on which a low-power modular multisensor node has been integrated. The system incorporates environmental sensors, including TH, particulate matter, and different gases. A movable component that rotates the sensor panel toward the wind direction has been designed. This rotation is controlled by a system based on an anemometer, which detects the wind direction and positions the panel accordingly. Connectivity with the central data platform has also been evaluated using two communication technologies: the long-range low-power LoRaWAN protocol and WiFi communication, enabling remote data transmission, automatic synchronization, and real-time integration with the digital twin platform.

At the hardware level, component selection and integration have been carried out according to the criteria of low energy consumption, lightweight design, and modularity. On the software side, embedded programming has been developed to enable synchronized acquisition from multiple sensors, edge preprocessing, and data packaging and transmission management. In addition, preliminary tests have been conducted in real-world environments to validate the feasibility and stability of the system in representative CH scenarios.

**Figure 9:**
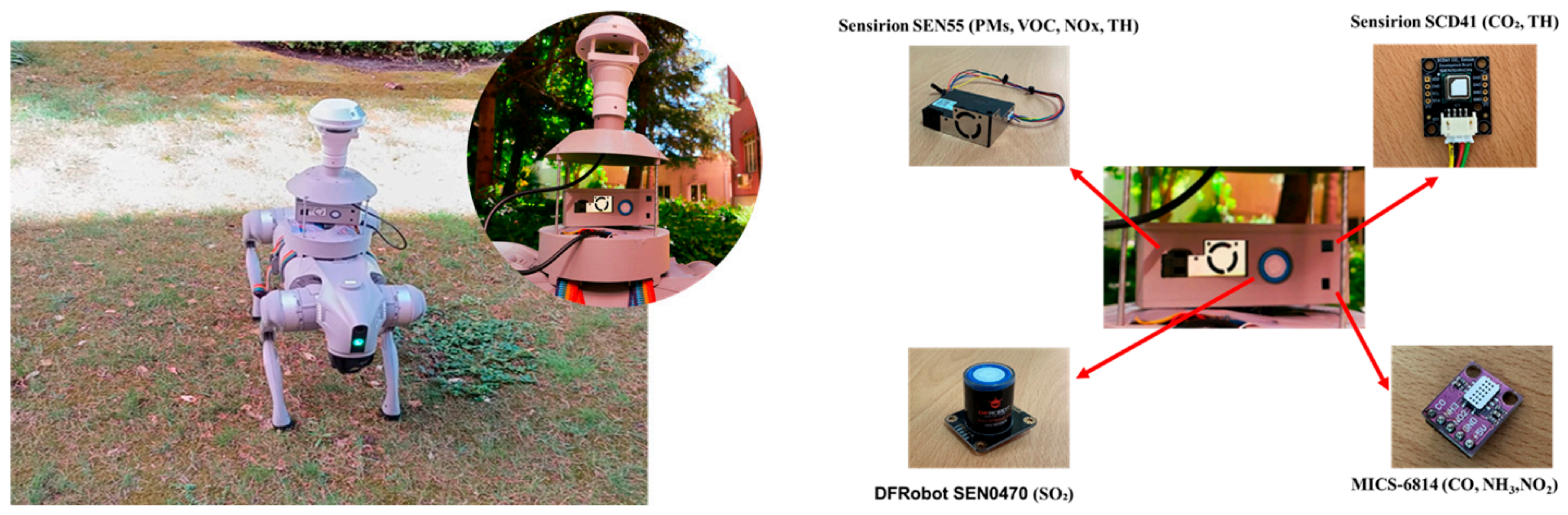
Prototype gas-sensing system with direction detection mounted on the Unitree GO2 robot (**left**) and monitoring system with the gas sensors used in this application (**right**).

Figure 9 shows the gas-sensing system (SO_2_, CO_2_, CO, NH_3_, NO_2_, PM_2.5_, temperature, and humidity) with direction detection mounted on the Unitree GO2 robot (Hangzhou Unitree Technology Co., Ltd., Hangzhou, China).

**Figure 10:**
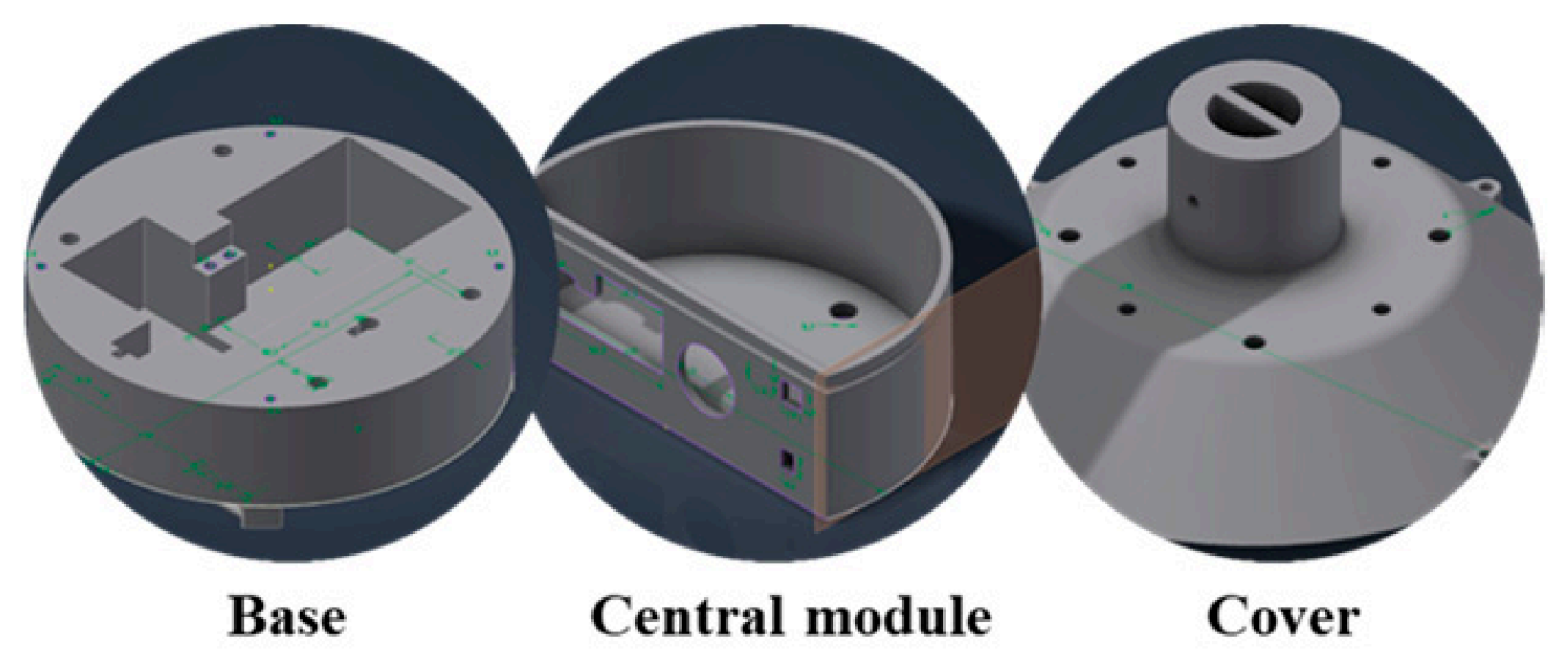
Mechanical design of the mobile platform.

The mechanical design of the system is composed of 3 modules: base, central module and cover, see Figure 10. In the base is where the rotation system is placed, in the central module the monitor system and in the cover is where the anemometer is placed. When a gas measurement has to be performed, the system measures the wind direction and it orientates the central module to this direction to measure the gas pollution.

In the central module is where the monitoring system is located and it is composed of an Arduino microcontroller with several gases sensors; see Figure 9 right. This module is in charge of sending the gas measurements using WiFi or LoRaWAN to the cloud.

A first version of this prototype was tested in Baltanás. The preliminary results obtained with this system have been presented in [25].

The robot can autonomously move through the monitored heritage environment while continuously acquiring environmental measurements at different locations. Compared with fixed wireless nodes, this mobile platform increases the spatial coverage of the monitoring system and enables inspections in areas where permanent sensor installation is difficult or undesirable. The rotating sensor panel further improves measurement quality by automatically orienting the sensors toward the airflow direction during operation.

## 4. Results

In this section, the preliminary results obtained with the different tailor-made systems are presented, demonstrating their capability to monitor key parameters and provide valuable information on the condition of CH sites, as well as to support decision making for their preservation.

### 4.1. Indoor Movement and Airflow Monitoring System Preliminary Results

**Figure 11:**
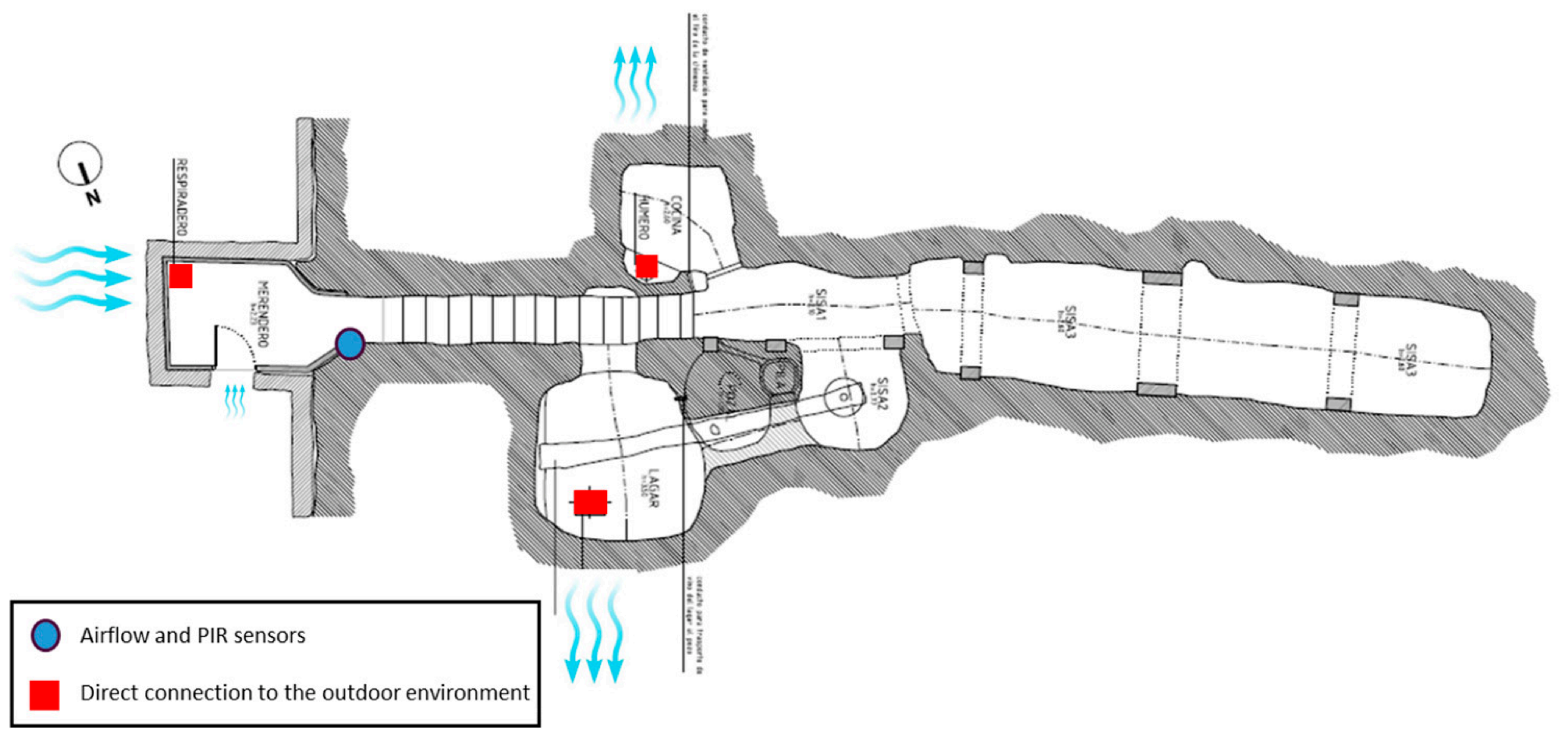
Cellar map with the point where airflow and PIR system has been deployed, illustrating the direct connections to the outdoor environment; the airflow direction within the cellar is inferred from the measurements obtained [30].

**Figure 12:**
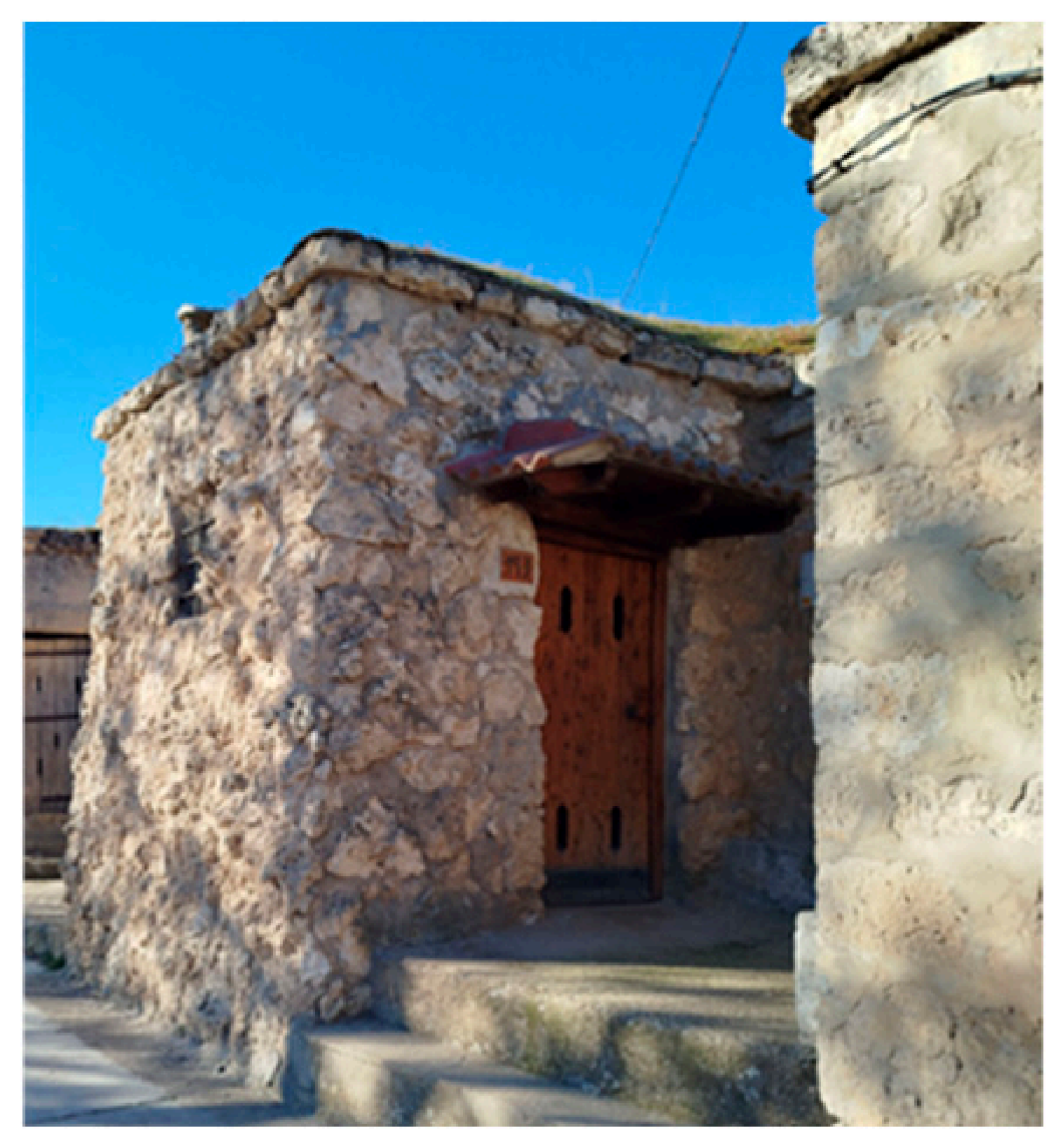
Picture of the main entrance of the cellar where the door and the lateral window can be seen.

In Figure 11 the map of the cellar indicating where the system to measure PIR and airflow has been placed is marked. In Figure 12, a picture of the main entrance of the cellar is shown. It should be noticed that there is an open window at the entrance and the door also has some holes for good ventilation.

**Figure 13:**
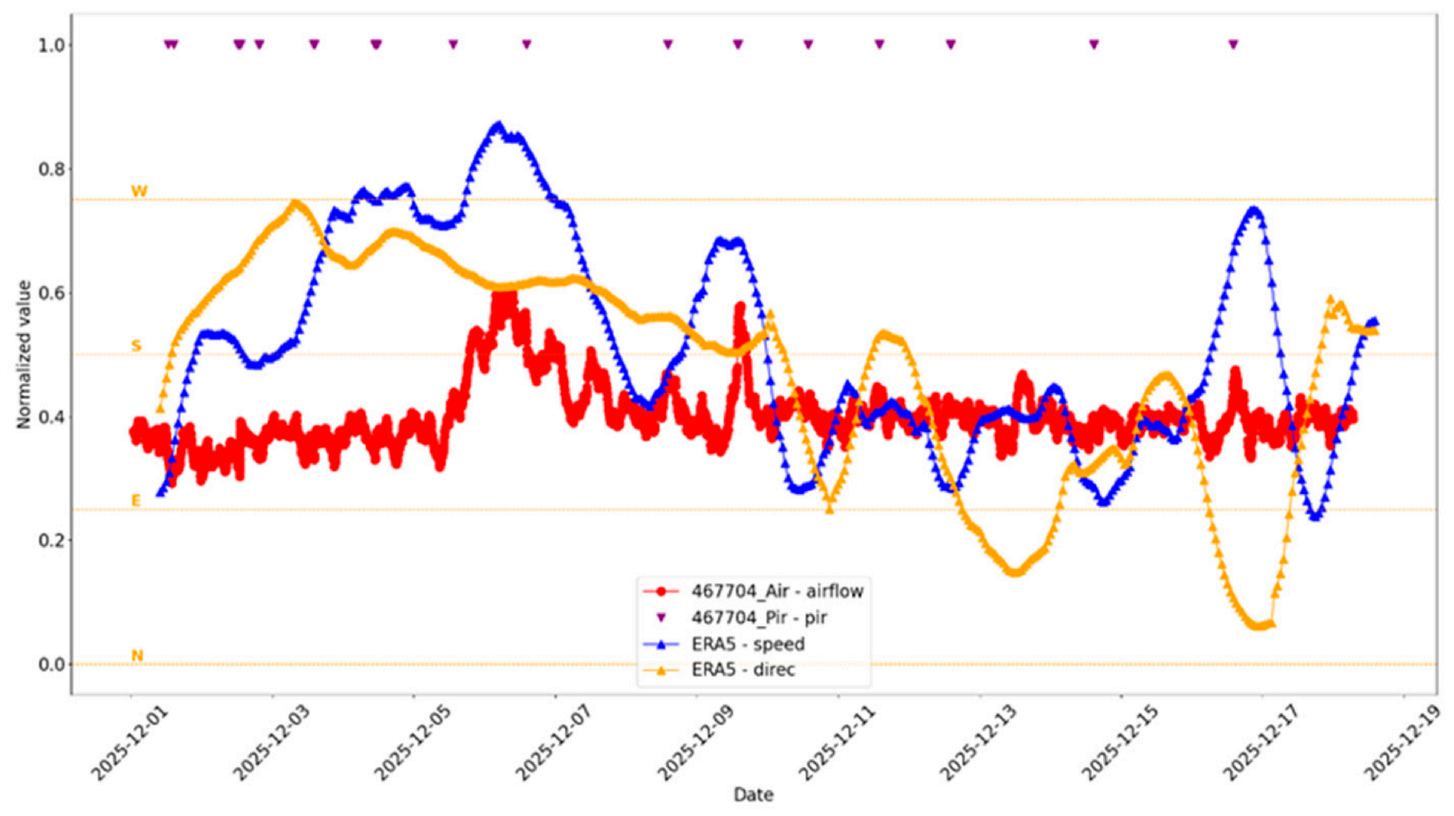
Airflow and PIR detections measured inside the cellar and wind speed and wind direction measured outside obtained from the Open-Meteo Historical Weather API, using the ERA5-Land reanalysis dataset provided by the European Centre for Medium-Range Weather Forecasts [31].

The preliminary results from one of the cellars are shown in Figure 13. In this figure the airflow measurement with the PIR sensor in the interior of the cellar close to the main door is compared with the exterior wind and direction velocity obtained from the Open-Meteo Historical Weather API, using the ERA5-Land reanalysis dataset provided by the European Centre for Medium-Range Weather Forecasts [31]. It can be seen that the velocity measured with the airflow sensor in the interior follows the same trend as the wind velocity measured at the exterior in the ERA dataset. The values shown in the figure are normalized but it should be noticed that the airflow values of the interior are always greater or equal to 0 m/s, meaning that the ventilation is coming from the window or the main door. It can also be seen that when the wind direction is close to the north, the velocity in the interior does not increase very much due to the orientation of the cellar. It was also noticed that when somebody enters the cellar and the wind velocity outside is high, a sudden increase can also be noticed in the interior, but the main ventilation occurs at the window close to the entrance.

In Figure 11, the three points in the cellar that have a direct connection to the outdoor environment are shown. The direction of airflow within the cellar is also inferred from the measurements performed. To validate this hypothesis, two additional airflow sensors should be installed at the other outdoor connection points.

**Figure 14:**
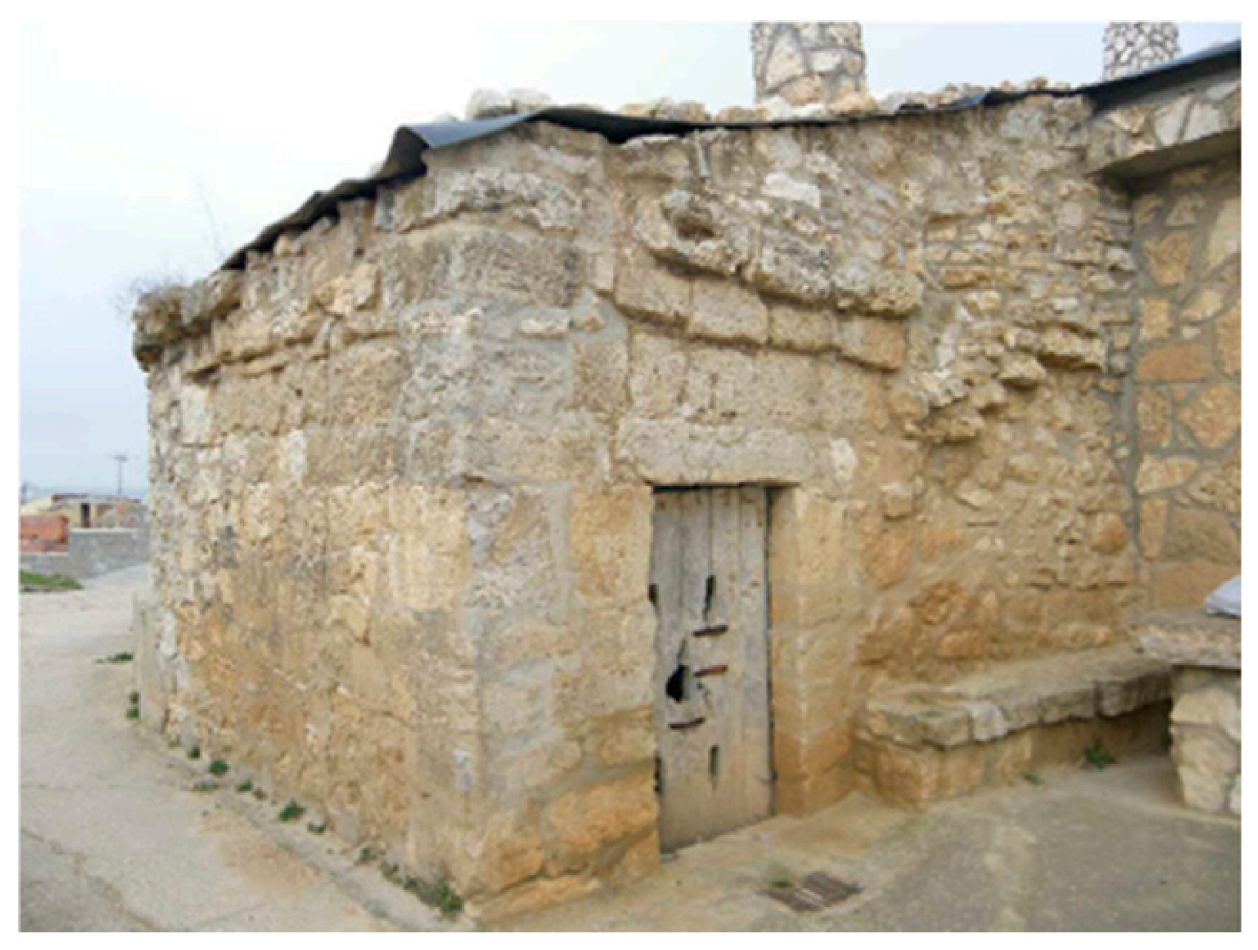
Picture of the main entrance of the second cellar where the door can be seen.

**Figure 15:**
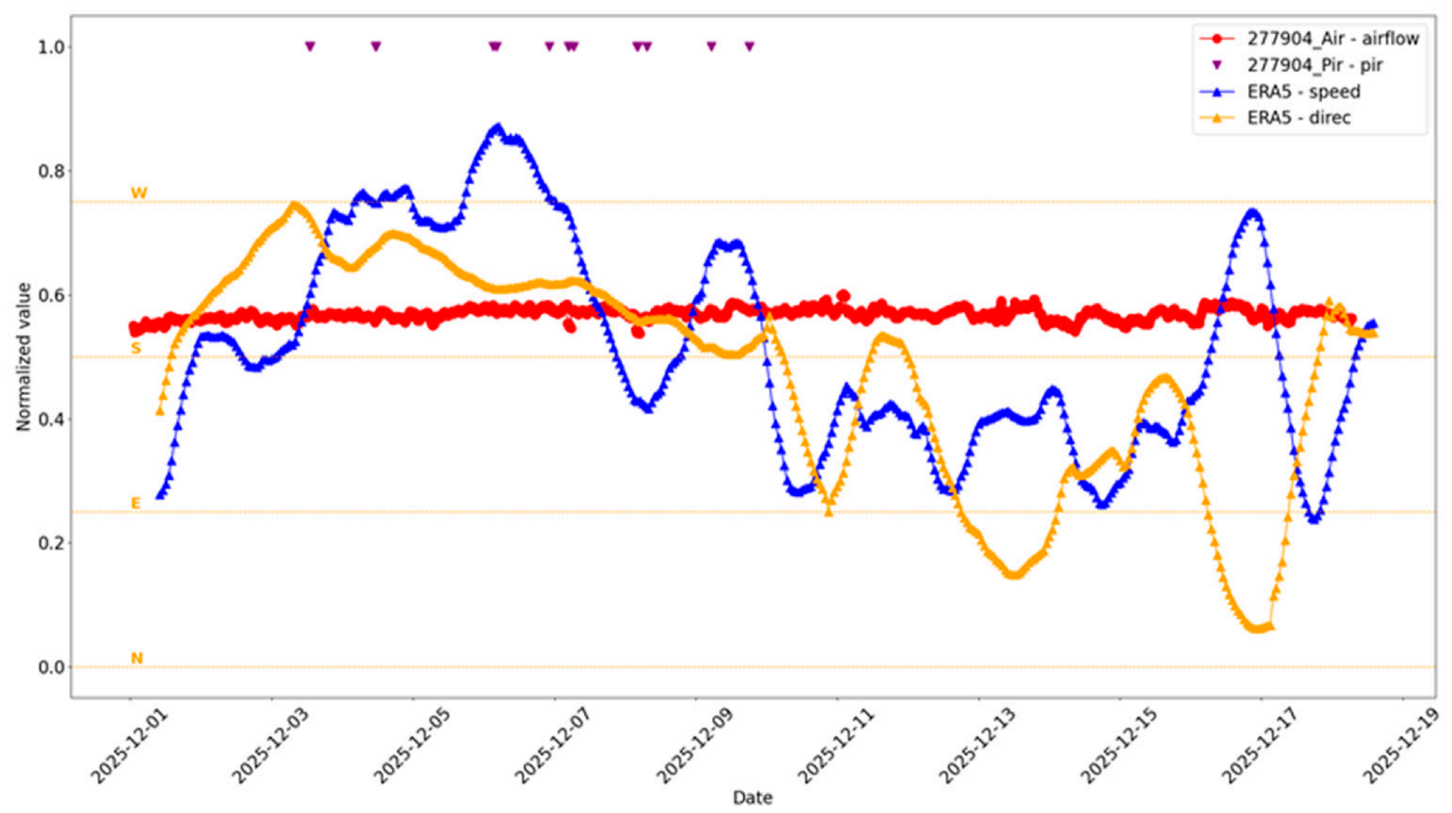
Airflow and PIR detections measured inside the cellar and wind speed and wind direction measured outside obtained from the Open-Meteo Historical Weather API, using the ERA5-Land reanalysis dataset provided by the European Centre for Medium-Range Weather Forecasts [31].

In the other cellar without that window, see Figure 14, the airflow sensor measures values lower than 0 m/s and this is because the ventilation goes in the other way around, see Figure 15. The air is going from the interior to the exterior through the main door.

**Figure 16:**
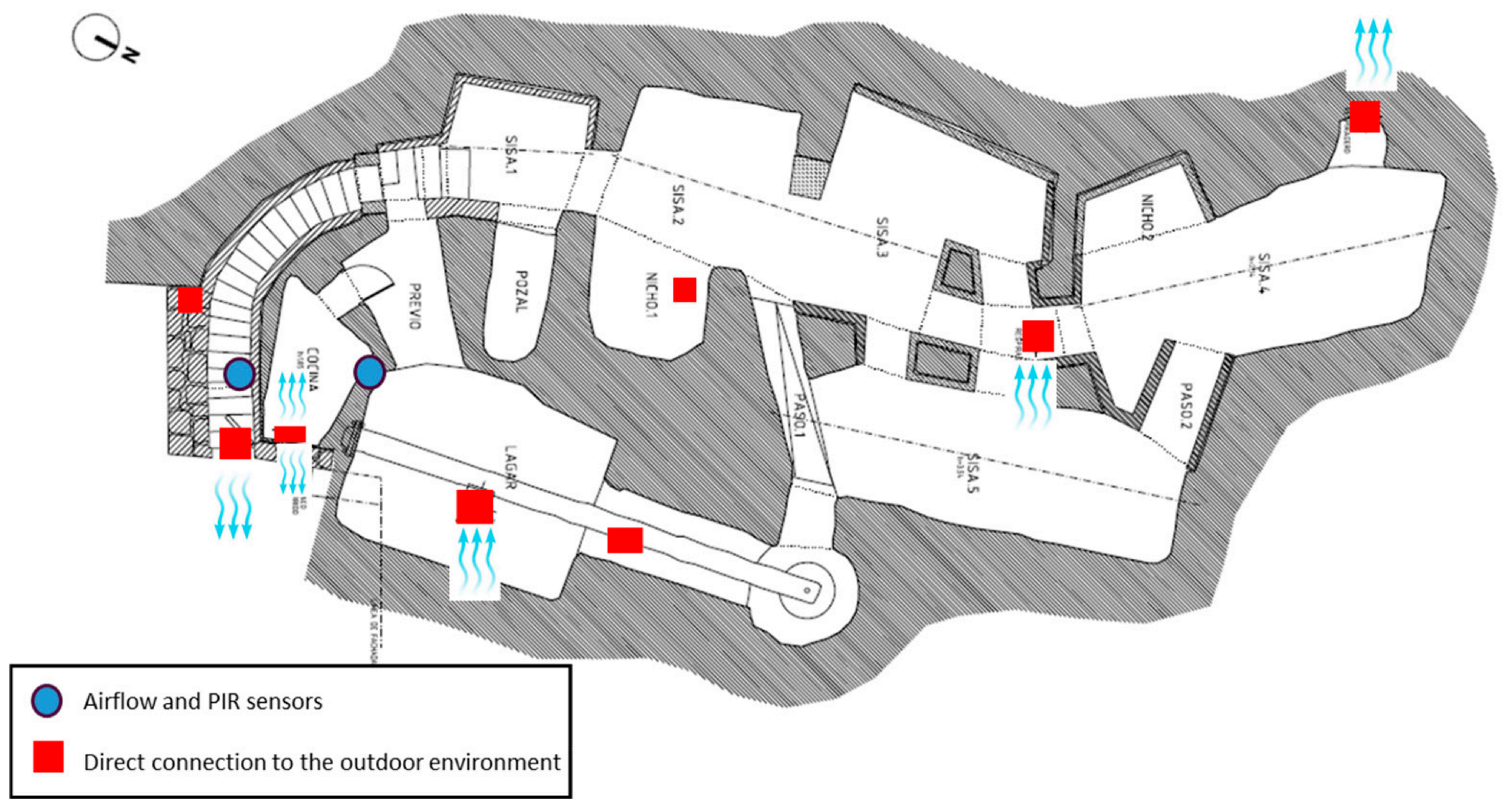
Map of the cellar with direct connections to the outdoor environment are shown and direction of the air in the cellar is also supposed by the measurements obtained [30].

To corroborate these results, four airflow sensors were installed at each connection to the exterior. The preliminary results are shown in [24]. The results show that airflows outward through two of the openings and inward through the other two, while, at one additional opening, the airflow direction varies, sometimes entering and sometimes exiting; see Figure 16.

### 4.2. Outdoor Visitor and Vehicle Monitoring System Preliminary Results

**Figure 17:**
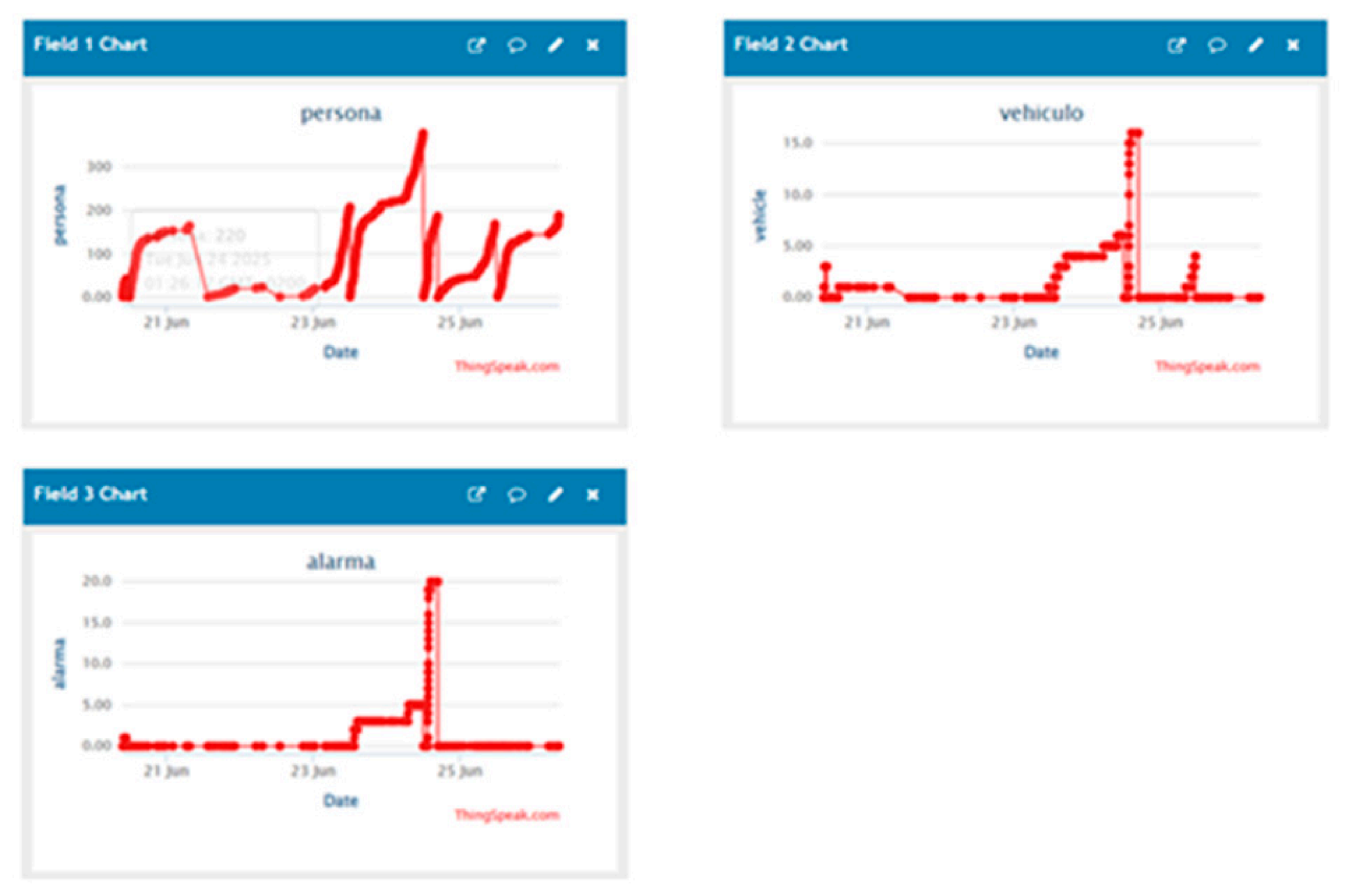
Data collected from visitor monitoring system (V1) visualized in the ThingSpeak platform.

Before deploying these systems in the Baltanás cellars, they were tested at ITEFI. Figure 17 shows part of the data stream transmitted in real time via the LoRaWAN network to the ThingSpeak cloud platform during the deployment of the V1 visitor monitoring node. Since the test took place in an indoor corridor with frequent pedestrian traffic, all detected objects were real pedestrians rather than vehicles. The figure presents three data curves. Field 1 (person) represents the cumulative number of detected pedestrian crossings. The curve displays a clear upward trend during daytime working hours—such as between 9:00 and 11:00 and between 15:00 and 18:00—which corresponds to the busiest periods of daily activity at the institute (ITEFI). This behavior demonstrates the strong detection sensitivity and operational stability of the system in real-world use. Field 2 (vehicle) is intended to indicate the number of vehicle crossings. However, because no actual vehicle activity occurred in the test area, all recorded counts must be considered false positives. These are likely caused by pedestrians walking slowly through the detection zone or briefly lingering within it—for example, cleaning staff with carts—exceeding the 1.5 s threshold for vehicle recognition and consequently being misclassified as vehicles. Field 3 (alarm) represents alarm events triggered by abnormally long beam obstructions lasting more than 10 s. This curve remains generally stable, with only small peaks during specific periods. On-site observation confirms that these peaks correspond to cases where people stopped in front of the sensor to talk, temporarily blocking the infrared beam.

All three curves show a sudden drop or discontinuity at roughly the same point each day (around 13:00). This effect is not a system anomaly but rather the result of the programmed reset mechanism built into the firmware. To ensure long-term operational stability in unattended environments, the V1 firmware triggers an automatic reset every 24 h, freeing system resources and refreshing communication states. This prevents issues associated with prolonged operation, such as memory leaks or communication module freezes. Additionally, if consecutive LoRaWAN uplink transmission failures exceed the preset threshold of five attempts, the system also activates the reset mechanism, initiating a forced self-recovery process. Each reset clears the counter values stored in the microcontroller’s memory, appearing as a step-like drop in the graph. This behavior is fully expected and intentionally implemented to enhance long-term reliability in unattended deployments.

**Figure 18:**
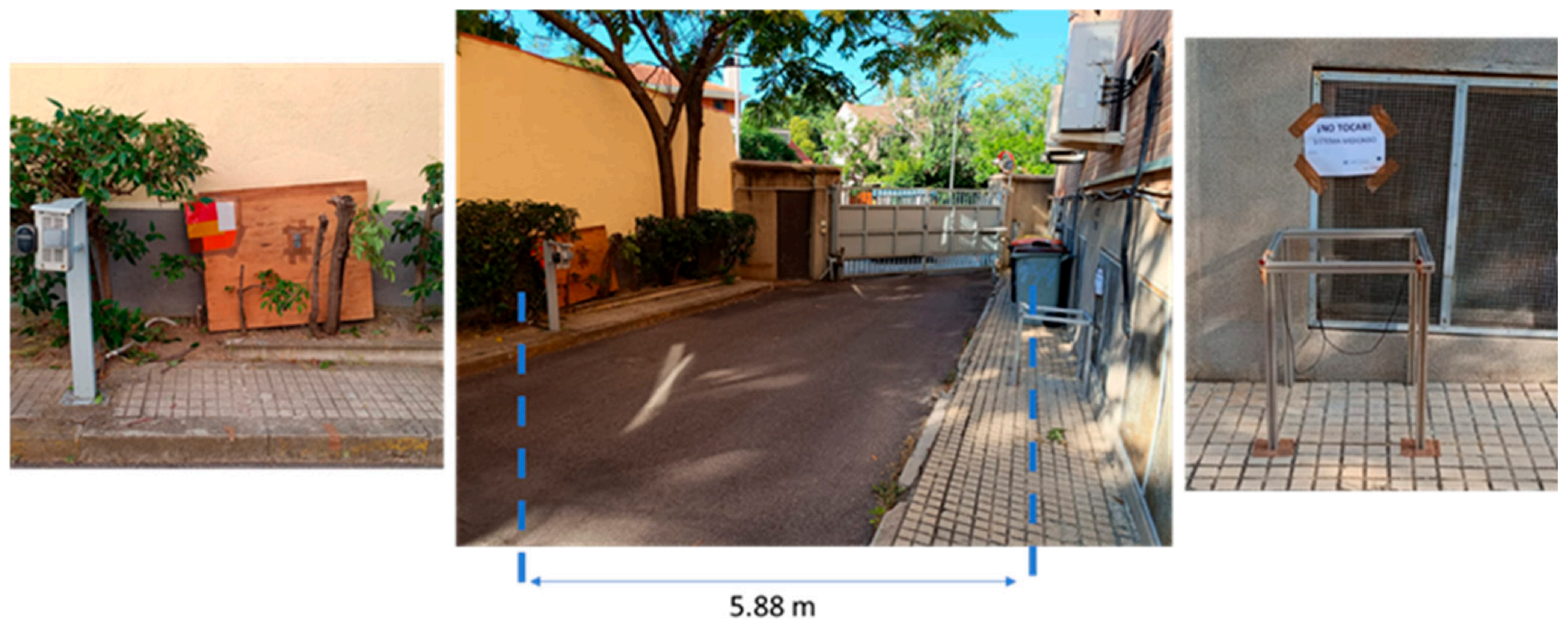
Visitor monitoring system (V2) deployed at ITEFI.

The V2 monitoring system was also tested at the facilities of ITEFI, as shown in Figure 18. In the center of the figure, the access road to the parking area can be observed. On the left, a wooden panel holding the reflectors is installed, while on the right, two reflective photoelectric sensors are mounted on an aluminum support structure.

**Figure 19:**
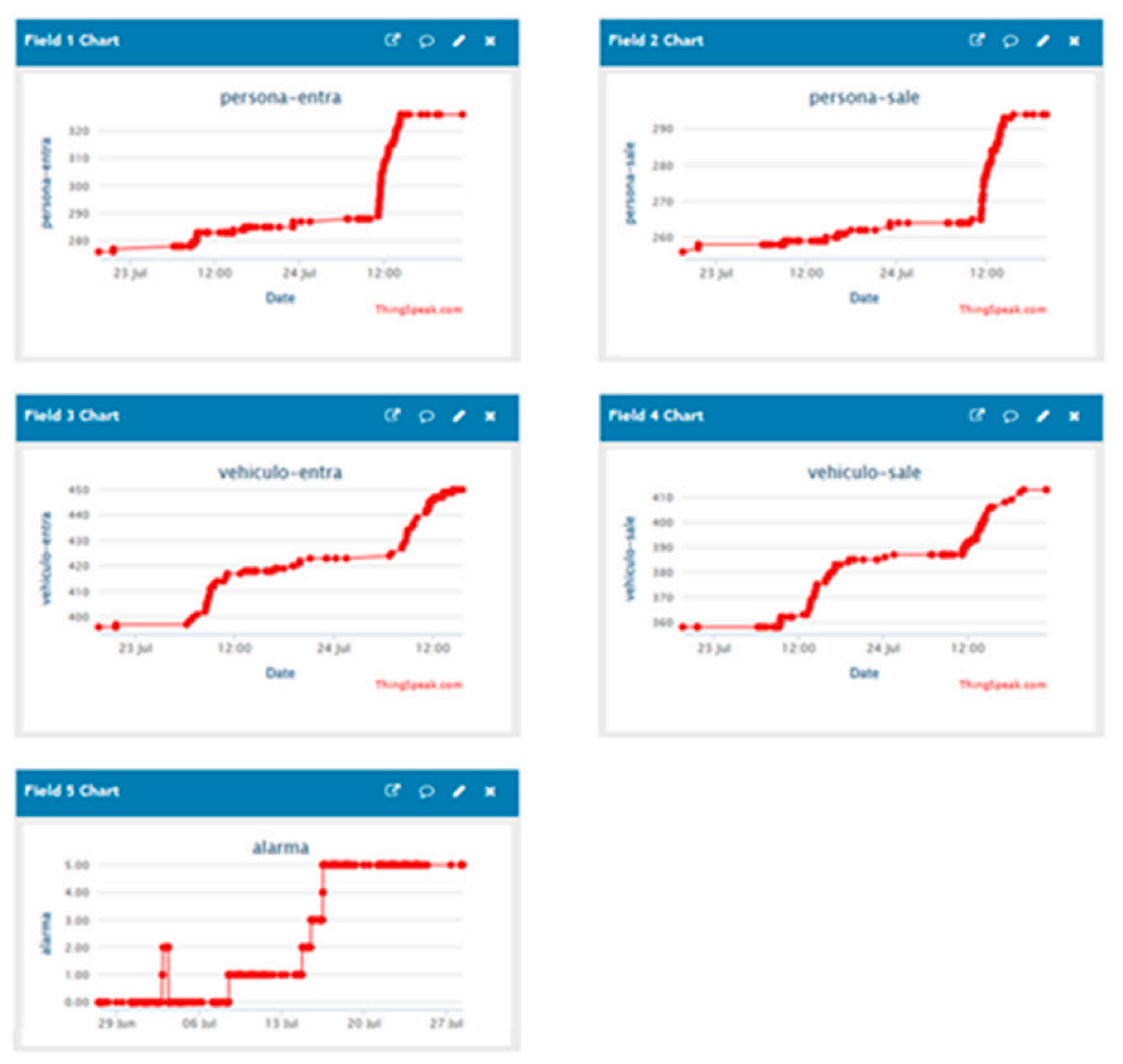
Data collected by the V2 system visualized in the ThingSpeak platform.

Figure 19 shows the data collected during a week for system V2. Field 1 corresponds to people entering, field 2 to people exiting, field 3 to cars entering, field 4 to cars exiting and field 5 to the alarm.

The information from Figure 19 can be further analyzed in detail in Table 3.

During the testing period, the system automatically recorded the daily number of vehicles and people entering and leaving the facility. Manual checks of the parking lot were also performed periodically to assess the accuracy of the system. Data for vehicle movements over one week were collected, showing daily entries, exits, and net flow.

The monitoring data clearly reveal cyclical patterns in parking occupancy frequency. Weekday traffic was significantly higher than weekend traffic, reflecting the real movement trends of staff. This demonstrates that the system can reliably capture dynamic changes in usage and maintain stable operation over time.

Counting accuracy was also high. Over the week, the system recorded 149 entries and 139 exits, with daily counts largely balanced. Minor discrepancies, such as zero entries and one exit on Sunday, are typical in long-term parking management, for example when vehicles remain parked overnight.

The net flow error of 10 cases in one week was not random. It was primarily caused by cleaning staff moving large waste containers near the prototype, which blocked multiple infrared beams and were mistakenly classified as vehicles. Excluding these identifiable interference events could raise the vehicle counting accuracy above 96%.

In conclusion, a single hardware upgrade from V1 to V2, adding a second sensor, along with software refactoring that introduced a finite state machine, systematically addressed all critical issues from the previous version. The result is a robust reliable visitor monitoring system that meets operational requirements.

As previously mentioned, since the direction of vehicle movement was not relevant for this analysis, two Version 1 systems were deployed in Baltanás. However, additional data are required to perform a comprehensive analysis and draw robust conclusions regarding the relationship between vehicle traffic and cellar vibrations.

### 4.3. TH and Soil Moisture Preliminary Results

**Figure 20:**
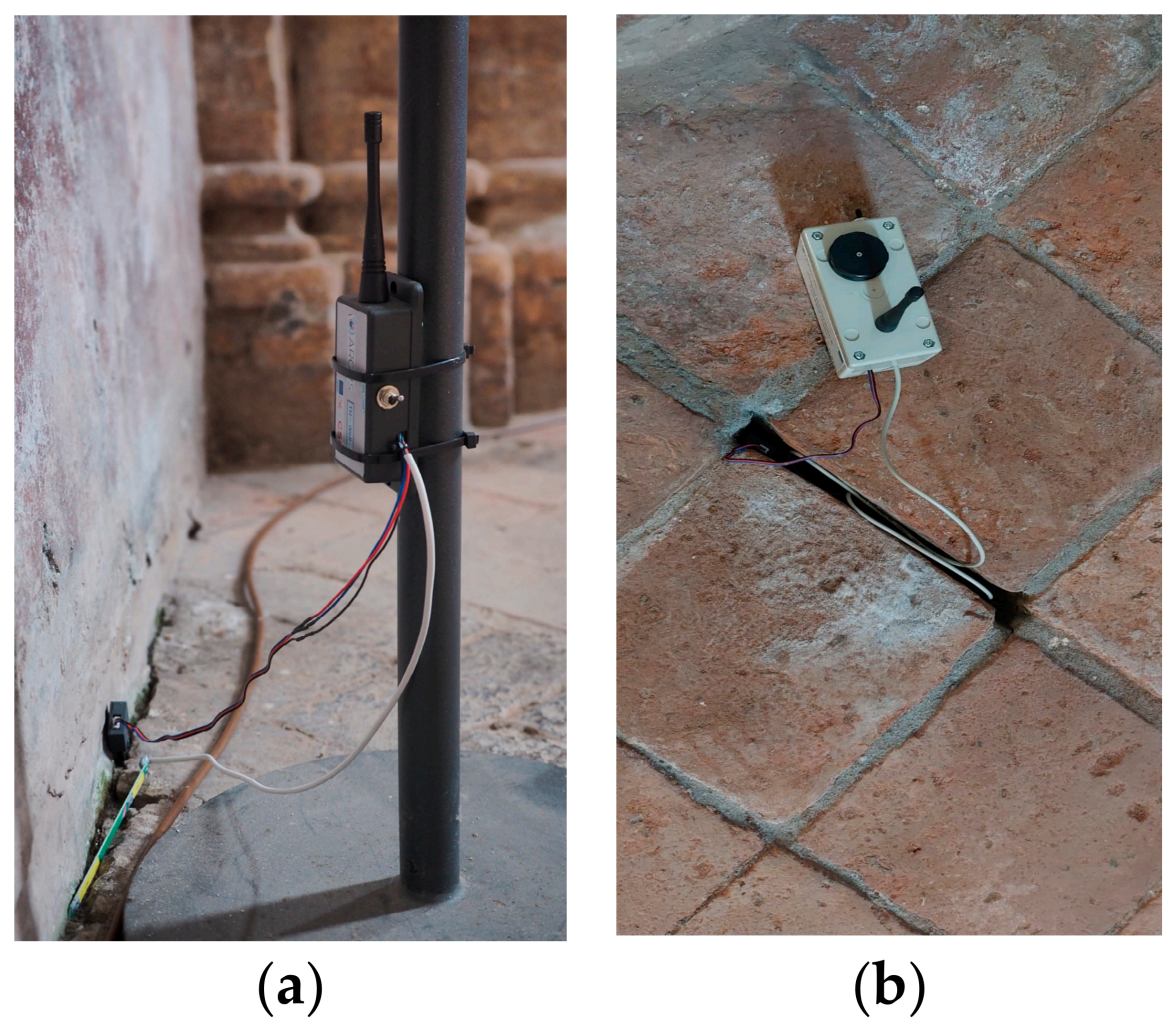
TH and soil moisture systems: (**a**) 3D6617; (**b**) 26680F.

Two systems (3D6617 and 26680F), each equipped with two sensors, one custom sensor developed by the authors to measure ambient temperature and humidity at two different depths, and one commercial sensor to measure VWC, were installed in two cracks in the Ranverso Abbey, see Figure 20. The 26680F system presented a malfunction, as the custom-developed sensor consistently recorded a value of zero.

**Figure 21:**
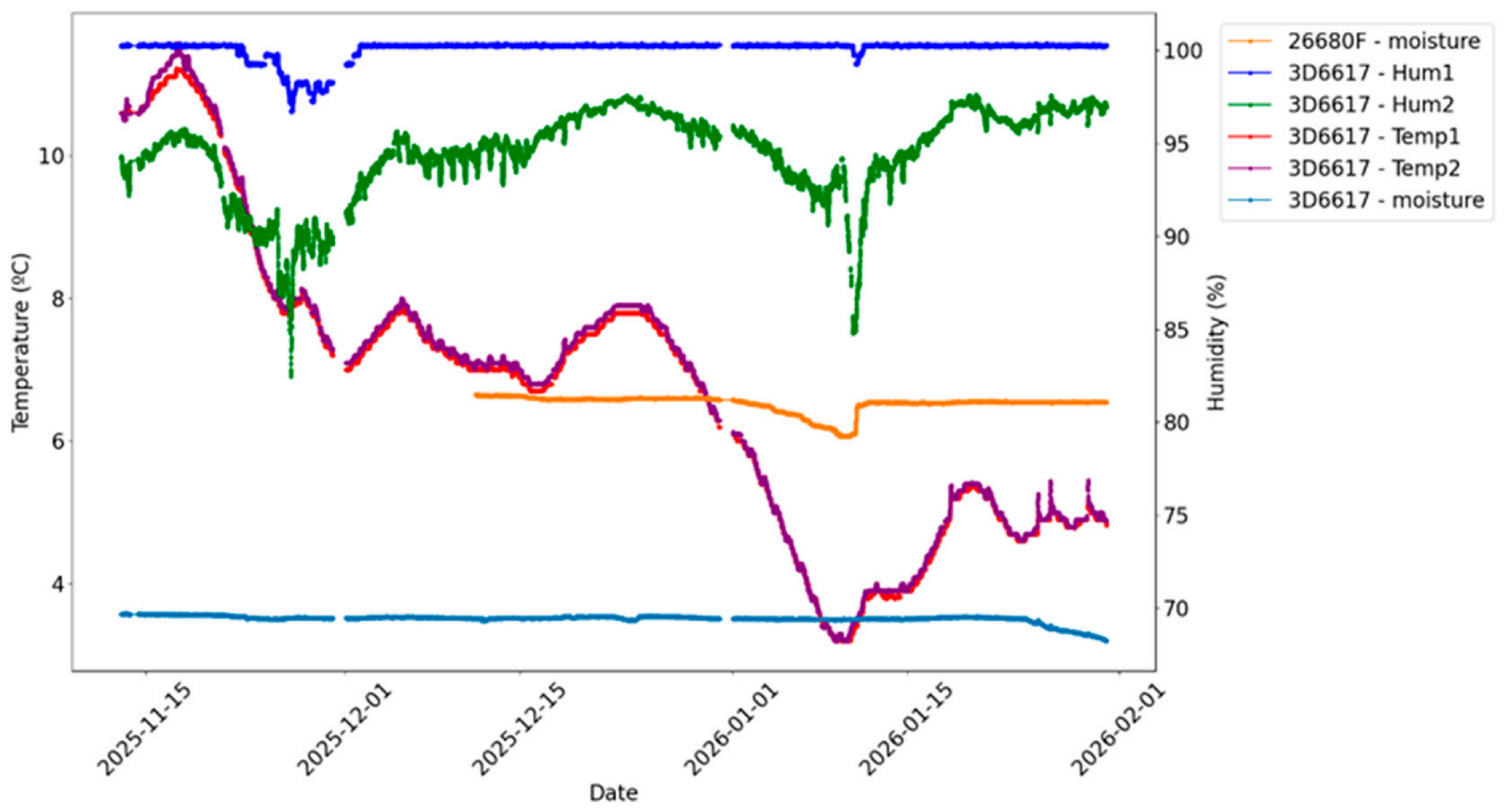
TH and soil moisture data obtained with both systems in Ranverso.

In Figure 21 the results obtained with these systems are shown. The temperature sensor Temp1 follows the same general trend as Temp2, including the significant drop observed in early January, indicating that both sensors are influenced by the same external conditions. However, Hum1 remains almost constant near 100%, which suggests possible sensor saturation. The sensor Hum2 behaves as a partially exposed microenvironment rather than a fully enclosed one. Hum2 remains consistently high (around 90–97%) but shows variability in response to temperature changes. This indicates interactions between ambient air and moisture stored in the wall. Overall, the crack is not completely isolated but experiences intermittent exchange with the exterior, resulting in a hybrid microclimate with elevated humidity and moderated temperature fluctuations.

The soil moisture sensors show very stable behavior, with only gradual changes over time and a slight decrease during the cold period in January. This is consistent with moisture retained in the material, where variations occur slowly and are governed by long-term processes. Additionally, the soil moisture and humidity sensors of system 26680F respond to the temperature drop, whereas the soil moisture measurements from system 3D6617 do not clearly capture this variation. Instead, they begin to decrease around mid-January, indicating a delayed response that is likely due to differences in installation conditions.

Overall, both temperature sensors respond to the same external forcing, the crack environment maintains high but variable humidity, Hum1 is likely compromised, and the soil moisture reflects a stable subsurface moisture regime with limited short-term variability.

### 4.4. Anemometer Preliminary Results

**Figure 22:**
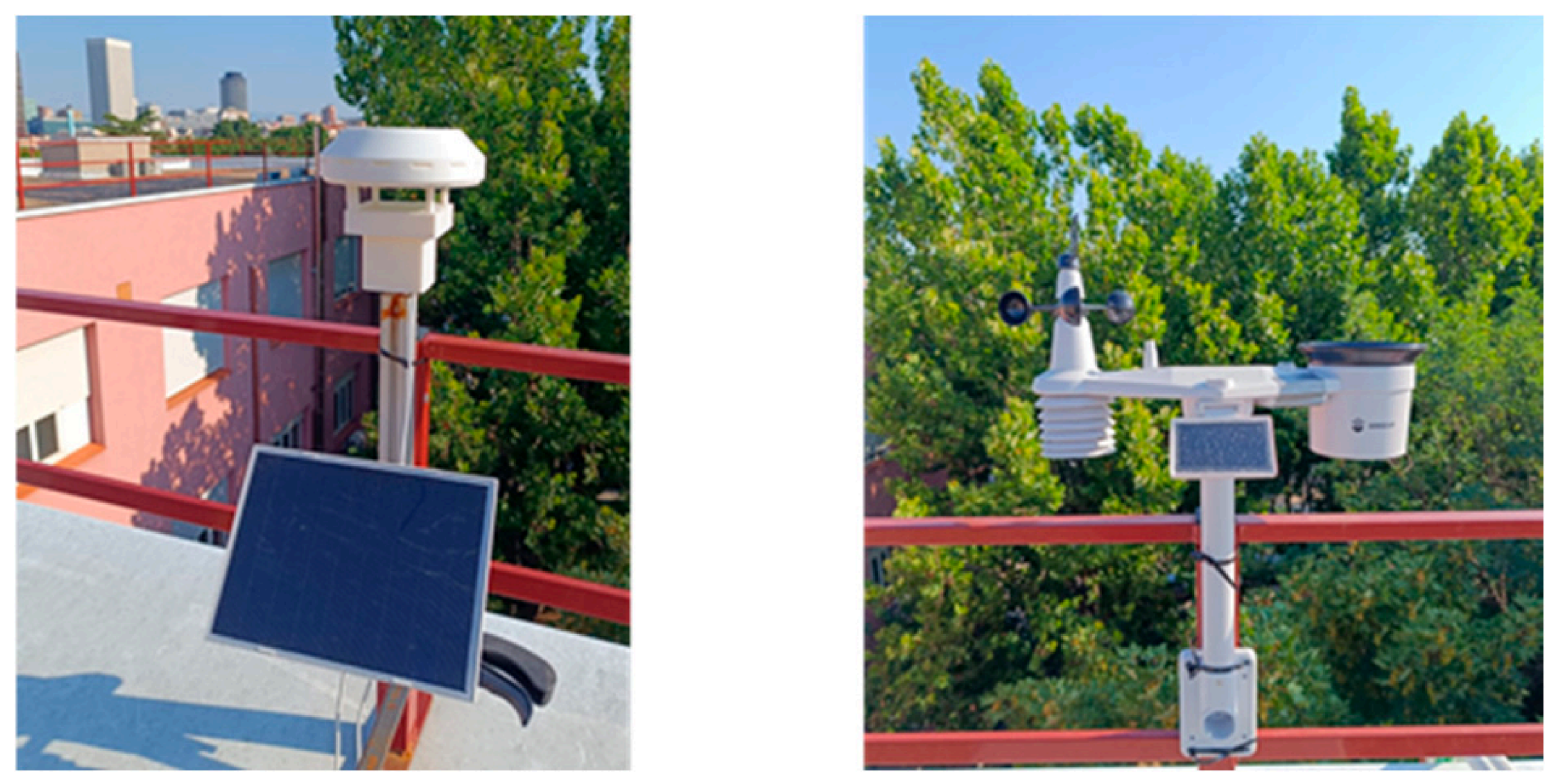
Anemometer based on MEMS sensors developed by the authors (**left**) and the commercial LoRaWAN weather station SenseCAP S2120 (**right**).

The anemometer based on MEMS sensors designed by the authors was compared with a commercial calibrated station LoRaWAN (SenseCAP S2120, Seeed Studio, Shenzhen, China); see Figure 22. This weather station is an 8-in-1 that measures air temperature, humidity, wind speed/direction, rainfall, light intensity, UV index, and barometric pressure. It features long-range connectivity and low power consumption (solar + batteries).

**Figure 23:**
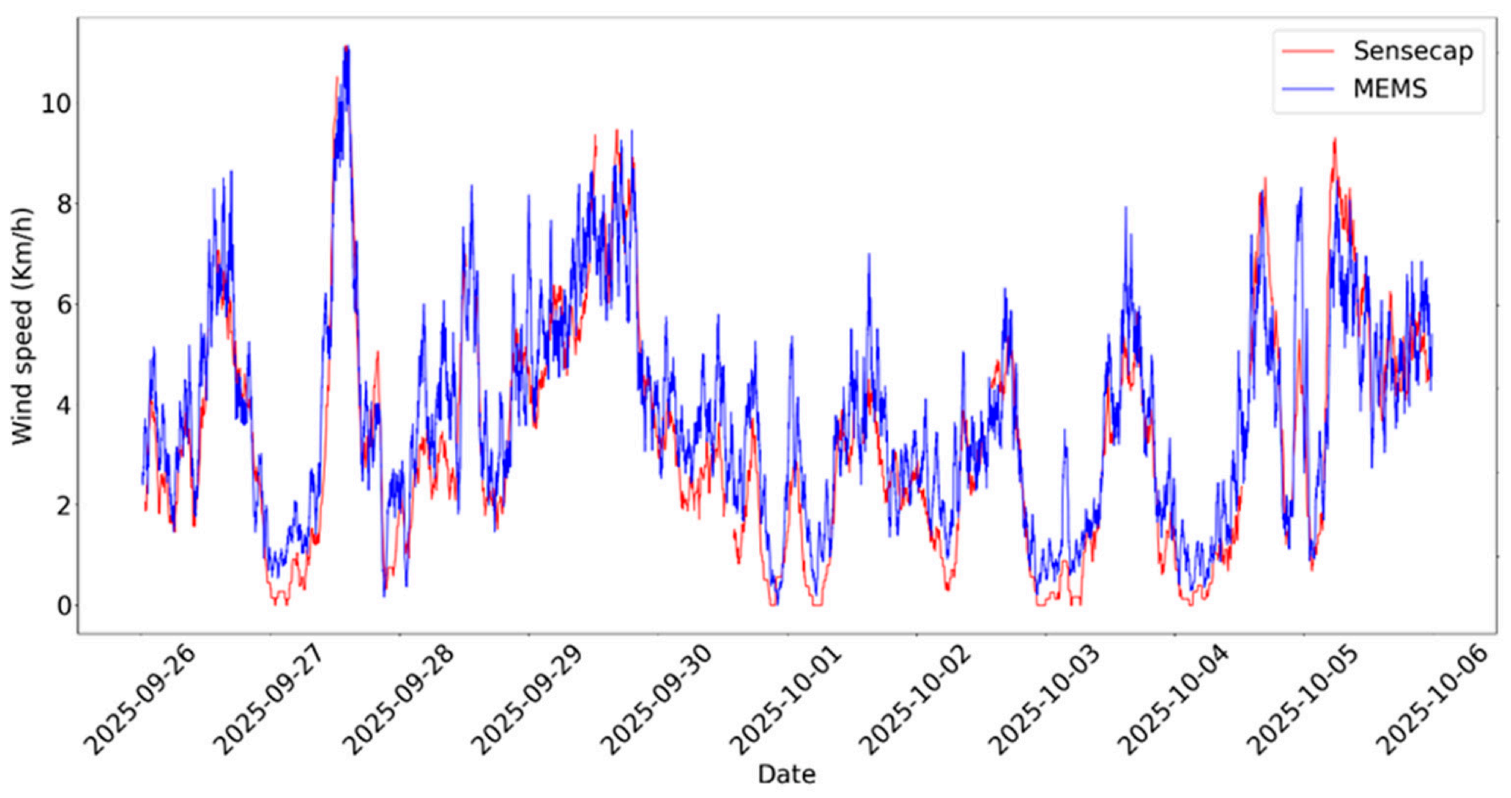
Wind speed results obtained from the MEMS anemometer developed by the authors and the commercial LoRaWAN weather station SenseCAP S2120.

**Figure 24:**
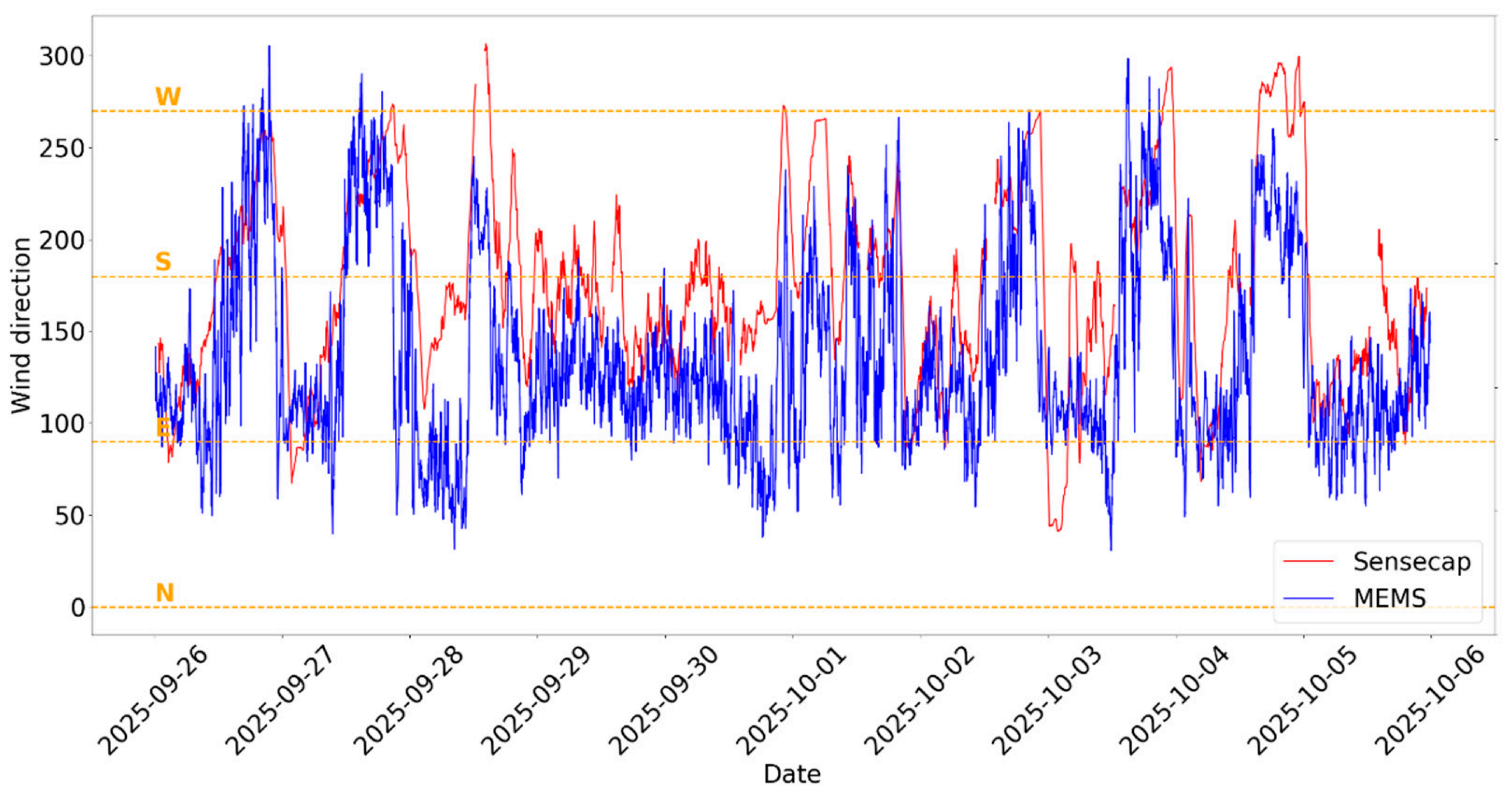
Wind direction results obtained from the MEMS anemometer developed by the authors and the commercial LoRaWAN weather station SenseCAP S2120.

The results obtained for measuring the wind speed and direction are shown in Figure 23 and Figure 24 respectively.

The MEMS prototype demonstrates strong potential for wind monitoring, showing good agreement with the calibrated SenseCAP station in wind speed dynamics and general directional trends. Nevertheless, it exhibits higher noise levels and reduced stability in wind direction estimation. These results suggest that further signal processing (e.g., filtering or averaging) would significantly improve its performance and make it suitable for deployment as a low-cost alternative sensing solution.

### 4.5. Pollution System Preliminary Results

**Figure 25:**
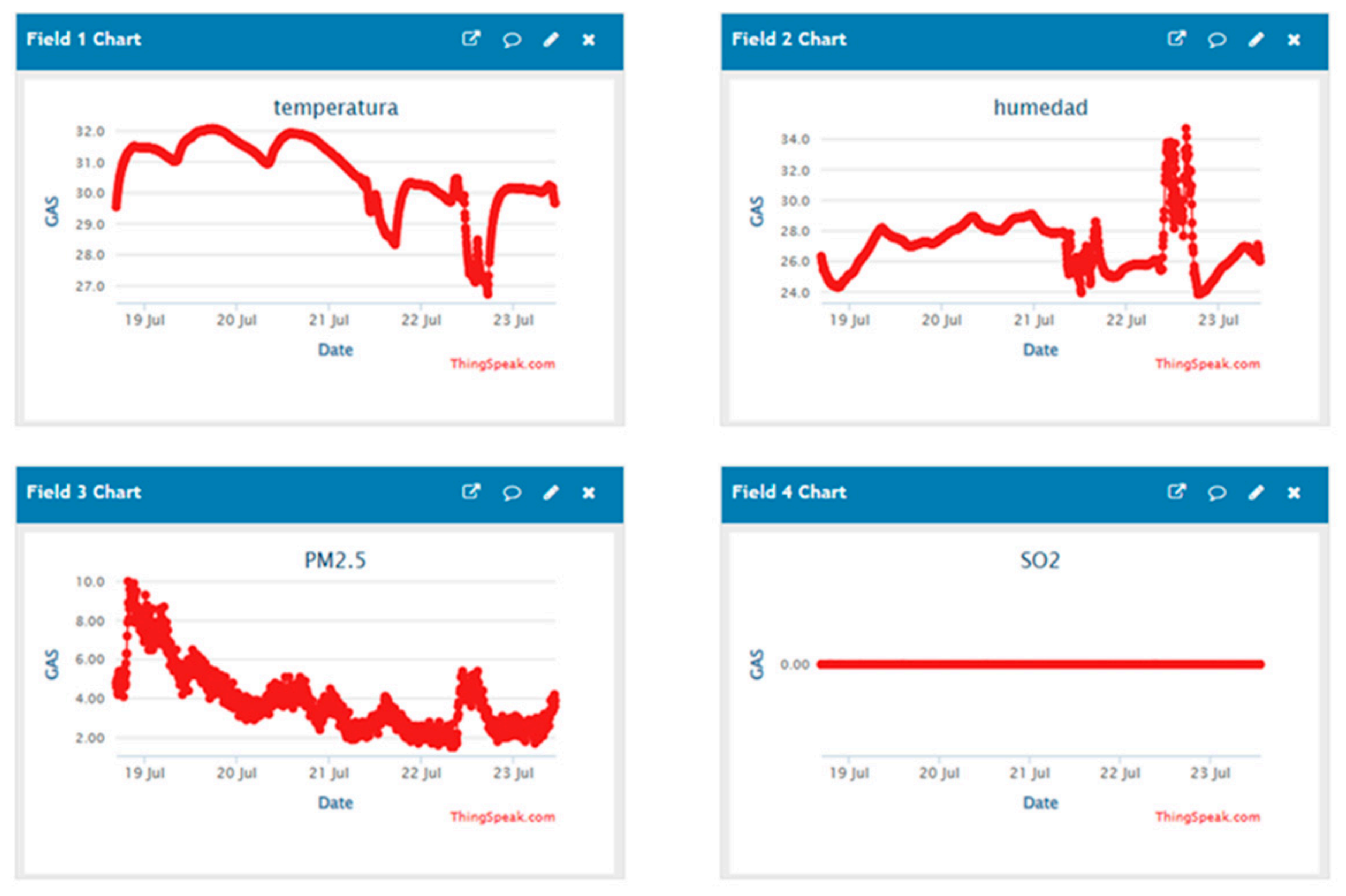
Temperature, humidity, particulate matter 2.5, and SO_2_ visualized in the ThingSpeak platform.

The system was tested for five days before being approved for deployment at Ranverso Abbey. In Figure 25, field 1 corresponds to temperature, field 2 to humidity, field 3 to particulate matter 2.5 (PM_2.5_) and field 4 to SO_2_.

**Figure 26:**
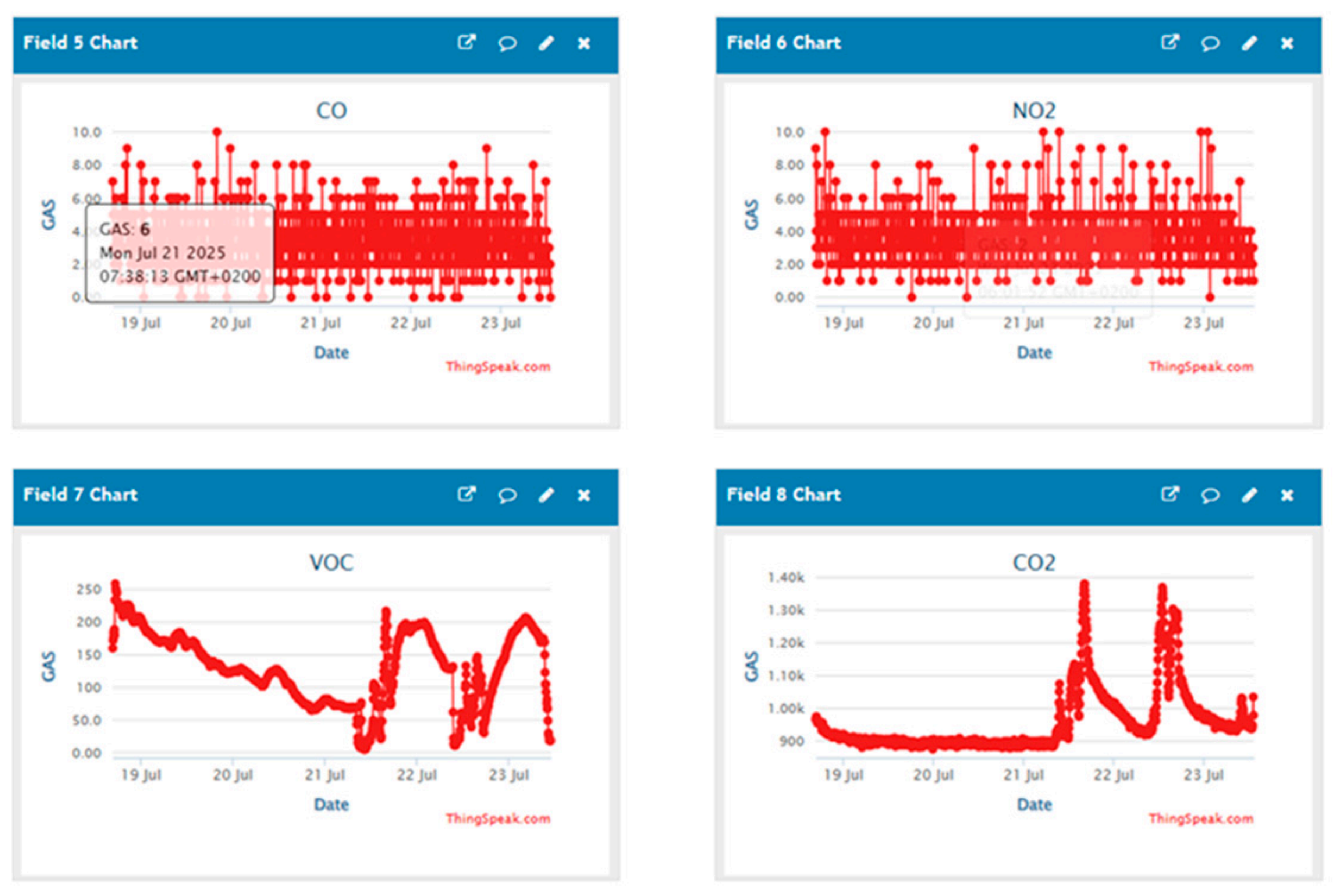
CO, NO_2_, VOC and CO_2_ visualized in the ThingSpeak platform.

Figure 26 shows the sensor readings of CO, NO_2_, CO_2_ and volatile organic compounds (VOC). Field 5 corresponds to CO, field 6 to NO_2_, field 7 to volatile organic compounds (VOCs) and field 8 to CO_2_. CO and NO_2_ are very noisy and the values are quite constant. CO_2_ measurements show the difference when there is a work day and when there is a free day; 19 and 20 July were Saturday and Sunday. Also, VOC decreases slowly during the weekend when the windows are closed and starts increasing on Monday.

**Figure 27:**
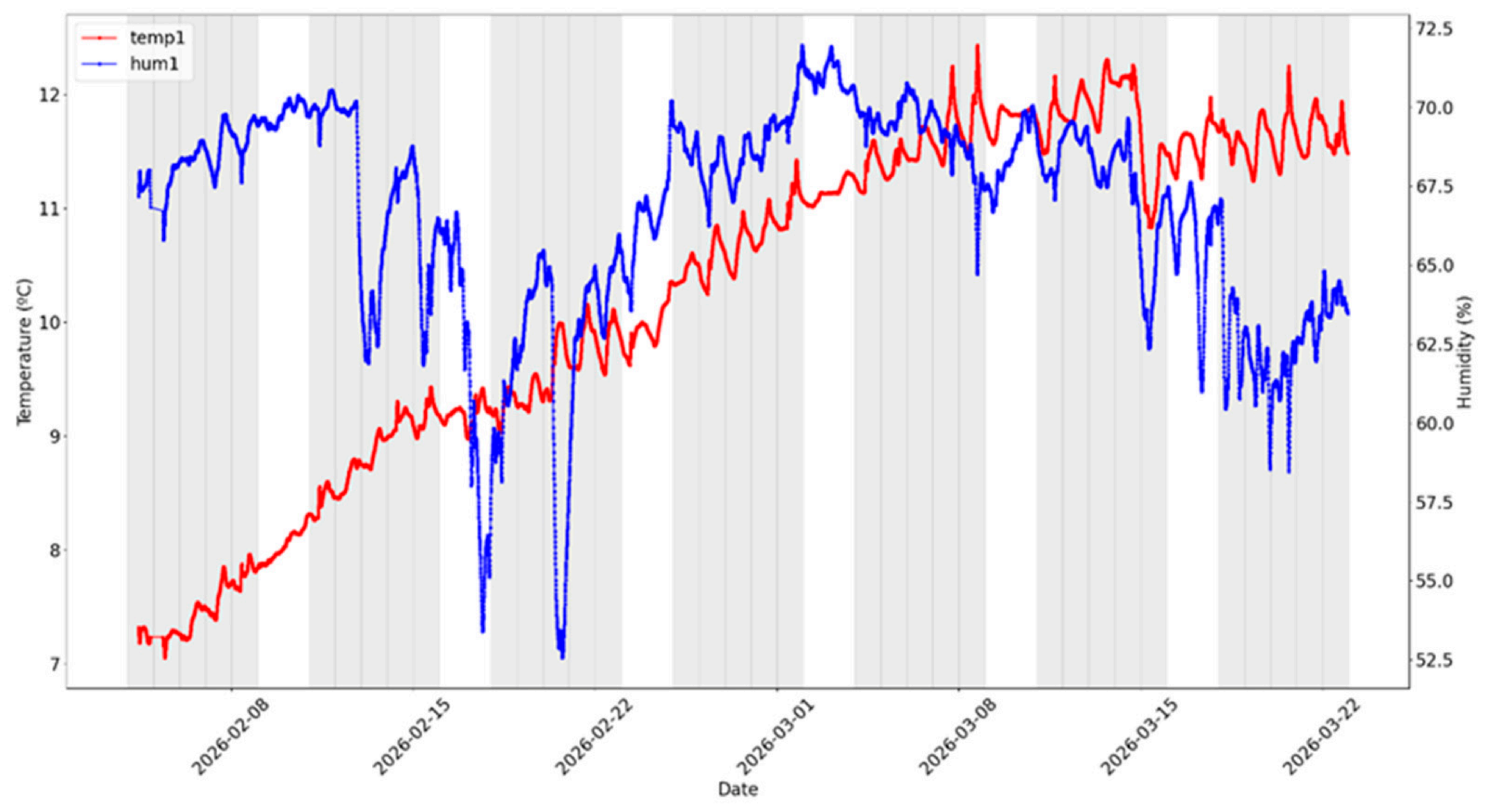
Temperature and humidity measured inside the abbey. In shadow is marked when the abbey is open to the public from Wednesday to Sunday.

**Figure 28:**
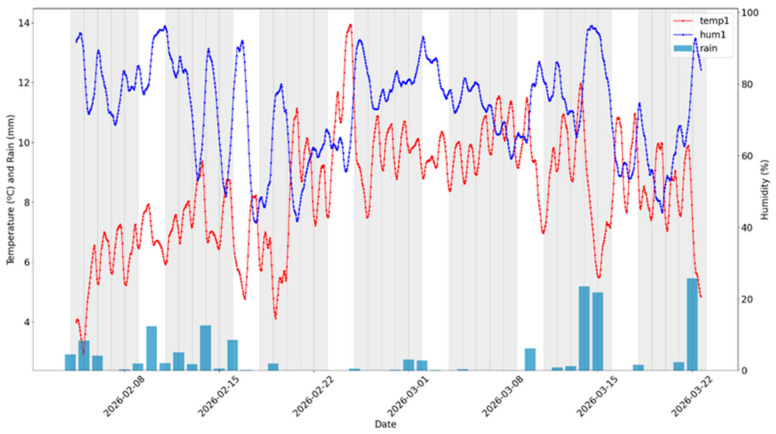
Temperature, humidity and rain obtained from the Open-Meteo Historical Weather API, using the ERA5-Land reanalysis dataset provided by the European Centre for Medium-Range Weather Forecasts [31]. In shadow is marked when the abbey is open to the public from Wednesday to Sunday.

In the next figures, the results obtained during almost 2 months in the Ranverso Abbey are shown. In shadow is marked when the abbey is open to the public from Wednesday to Sunday. In Figure 27 the temperature and humidity values in the interior of the abbey are shown. These values are compared with the meteorological data obtained from the Open-Meteo Historical Weather API, using the ERA5-Land reanalysis dataset provided by the European Centre for Medium-Range Weather Forecasts [31], Figure 28.

The comparison between indoor measurements and ERA5-Land reanalysis data highlights the strong hygrothermal buffering capacity of the abbey. While outdoor conditions exhibit pronounced diurnal variability and rapid responses to precipitation events, indoor temperature and relative humidity remain significantly more stable, with reduced amplitude and smoother temporal evolution. The absence of sharp humidity peaks indoors, even during rainfall events, suggests a delayed and attenuated moisture transfer through the building envelope. This behavior indicates the presence of high thermal inertia and moisture buffering capacity of the construction materials, which effectively decouple indoor microclimatic conditions from external atmospheric variability.

**Figure 29:**
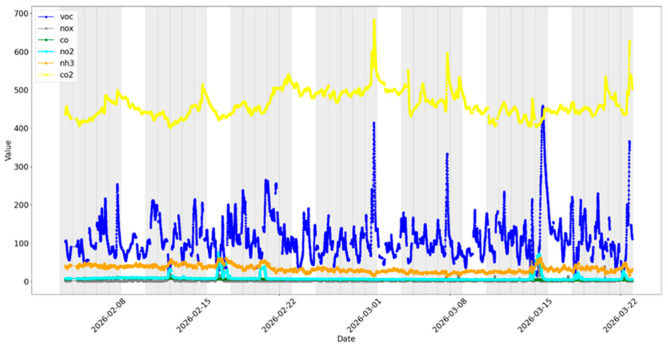
Gases measured in the interior of the abbey. In shadow is marked when the abbey is open to the public from Wednesday to Sunday.

In Figure 29 the different gases measured in the interior of the abbey are shown. The SO_2_ values are not shown since always were zero. The results show that the different pollutants analyzed are governed by clearly distinct emission sources and control mechanisms within the building.

First, CO_2_ exhibits a pattern consistent with occupancy. Concentrations increase during periods when the building is open to the public and reach higher sustained levels during weekend events. This confirms CO_2_ as a robust proxy for human presence and ventilation effectiveness.

In contrast, volatile organic compounds (VOCs) display highly episodic behavior, characterized by sharp short-lived peaks. Although these peaks occur more frequently during occupied periods and events, they do not scale linearly with occupancy. This suggests that VOC levels are primarily driven by specific short-term indoor sources, such as cleaning activities, use of products, materials, or particular event-related actions, rather than by occupancy alone.

**Figure 30:**
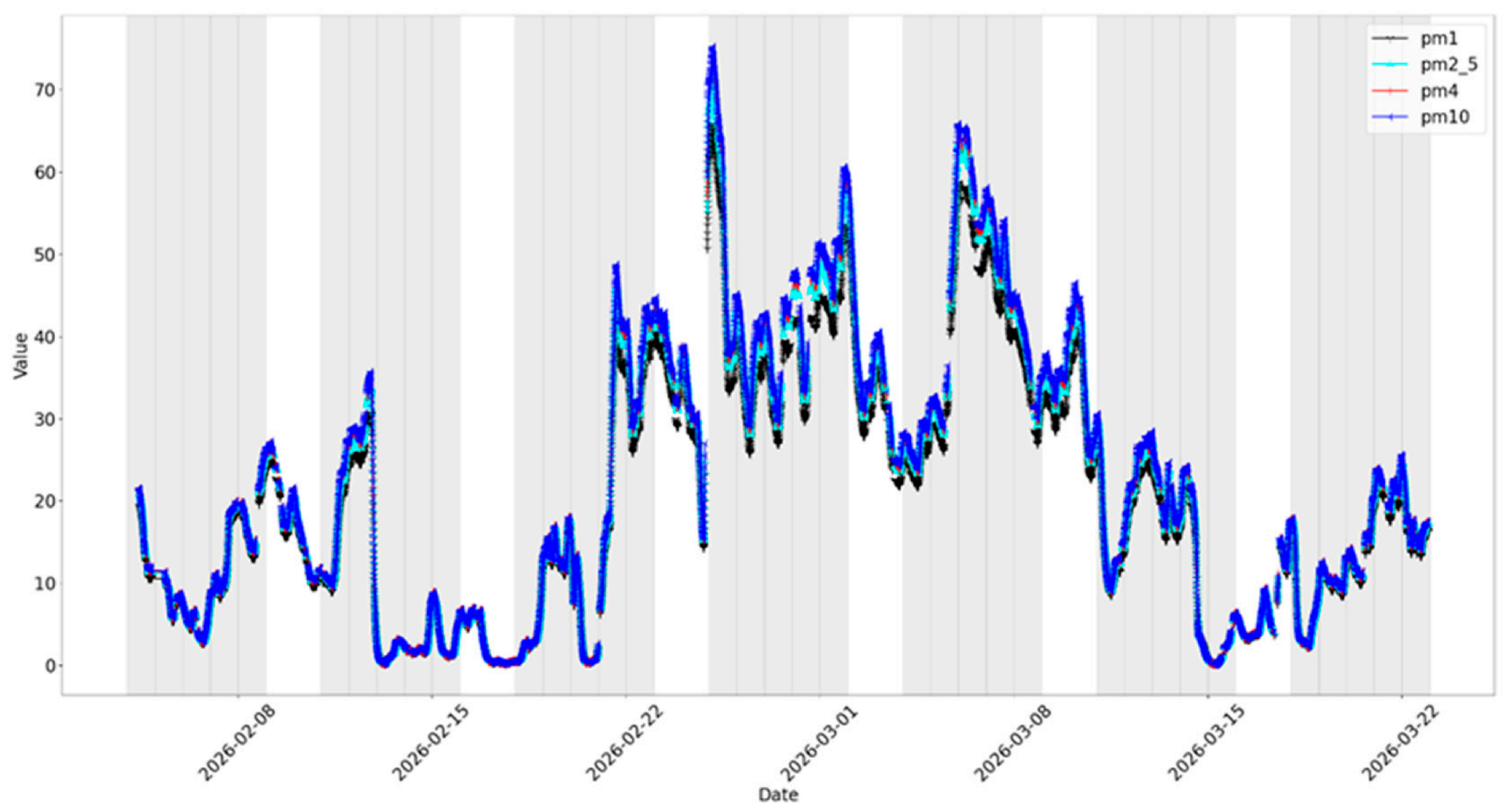
PM concentrations measured at the interior of the abbey. In shadow is marked when the abbey is open to the public from Wednesday to Sunday.

Regarding particulate matter (PM_1_, PM_2.5_, PM_4_, and PM_10_), all fractions show a smooth temporal evolution and are strongly correlated with each other, indicating a common and continuous source, see Figure 30. PM concentrations do not exhibit clear increases associated with opening periods or events and lack the abrupt peaks observed for VOCs. This pattern suggests that PM levels are mainly influenced by background conditions, likely of outdoor origin, and by ventilation and filtration processes, rather than by intermittent indoor activities. It should be noted that the abbey is very close to a highway that could affect the measurements.

Importantly, VOC peaks do not show a consistent pattern of co-occurrence with PM concentrations, indicating that the processes governing these pollutants are largely independent in the studied environment. While occasional simultaneous increases may occur, they are not systematic and do not support a direct causal relationship.

Overall, the findings indicate that indoor air quality in the building is controlled by three primary factors: (i) occupancy (reflected by CO_2_), (ii) specific indoor activities (VOCs), and (iii) background environmental conditions and air handling systems (PM). These results highlight the need for pollutant-specific management strategies, rather than assuming coupled behavior among different air quality indicators.

VOC concentrations do not show a consistent relationship with rainfall events. However, a moderate dependence on environmental conditions is observed, with higher concentrations generally associated with increased temperature and, to a lesser extent, high humidity levels. This suggests that VOC variability is primarily driven by emission processes and atmospheric conditions rather than wet scavenging.

The results show a clear decrease in particulate matter concentrations during rainfall events, confirming the effect of wet scavenging. However, high PM episodes also occur independently of precipitation, indicating the influence of additional atmospheric or emission-related factors.

### 4.6. Discussion of the Results

The experimental deployments carried out in the five pilot sites demonstrate that the developed monitoring framework can be successfully adapted to different CH scenarios, including historical buildings, archaeological sites, underground structures, and religious monuments. The results obtained from the different sensing systems confirm the feasibility of integrating heterogeneous sensing technologies within a common LoRaWAN-based communication architecture while maintaining low power consumption, long-range wireless communication, and non-invasive installation.

Recent reviews have highlighted the growing interest in IoT, wireless sensor networks, and smart monitoring technologies for preventive conservation of cultural heritage, demonstrating their potential for environmental monitoring and structural health assessment [9,10,25,26]. Previous studies have successfully applied wireless sensor networks to monitor historical buildings and archaeological sites [2,3,4,5,6,7,8], while more recent IoT-based frameworks have improved energy efficiency and remote monitoring capabilities [13,14,15,16]. However, most of these approaches focus on a single sensing application, such as environmental monitoring or structural health monitoring, or rely on proprietary platforms that limit interoperability and the integration of application-specific sensing technologies [9,10,15].

The development of the sensing systems presented in this work was motivated by the limited availability of commercial solutions capable of meeting the diverse monitoring requirements encountered in CH environments. Existing commercial monitoring plat-forms are generally based on proprietary architectures that restrict interoperability and the integration of customized sensing devices, making them difficult to adapt to heterogeneous heritage scenarios. In contrast, the proposed framework provides an open, modular, and scalable architecture that enables the seamless integration of multiple sensing modalities, including environmental monitoring, visitor counting, crack monitoring, airflow analysis, volumetric water content measurements, and mobile robotic inspection. Unlike previous works that primarily address individual monitoring applications [2,3,4,5,6,7,8,13,14,15,16], the proposed framework combines fixed wireless sensing nodes with UAV- and quadruped-based mobile sensing platforms within a common LoRaWAN communication architecture, providing both continuous monitoring and on-demand inspections of difficult-to-access areas. Although several IoT-based monitoring systems have been reported for cultural heritage applications, few studies have described the integration of fixed environmental sensing, visitor monitoring, structural monitoring, and robotic inspection platforms within a single LoRaWAN-based architecture validated across multiple cultural heritage pilot sites.

Furthermore, the validation carried out in five pilot sites distributed across four European countries demonstrates the adaptability of the proposed architecture to different environmental conditions, monument typologies, and operational requirements. This multi-site validation extends beyond the single-case studies commonly reported in the literature [2,3,4,5,6,7,8] and represents an initial step towards comprehensive smart monitoring frameworks integrating heterogeneous sensors, mobile robotics, and future digital twin technologies for preventive conservation [17,18,19,20,21].

The validation campaigns also provided quantitative evidence of system performance, as summarized in Table 4. The developed framework enabled the monitoring of more than ten environmental and anthropogenic variables, including temperature, relative humidity, airflow, wind speed and direction, moisture, particulate matter, gases, and visitor activity. Several monitoring systems operated continuously over multi-week to multi-month periods, demonstrating the capability of the developed architecture to support long-term data acquisition in real heritage environments. Among the evaluated sub-systems, the MEMS-based wind monitoring prototype exhibited a high temporal correlation with the commercial SenseCAP weather station for wind speed measurements (R = 0.904), while the visitor monitoring system achieved a vehicle counting accuracy exceeding 96% under controlled conditions.

From the perspective of preventive conservation, the proposed monitoring framework enables the continuous acquisition of environmental, structural, and visitor-related information, providing conservation managers with objective data to identify potentially harmful conditions before significant deterioration occurs.

The results presented in this work correspond to the initial validation phase of the monitoring systems under real operating conditions within the ARGUS project. While the current deployments demonstrate the feasibility and robustness of the presented approach, long-term monitoring campaigns are still ongoing and will provide larger datasets to further assess system performance, reliability, and long-term applicability. These future datasets will also support the integration of predictive maintenance and artificial intelligence techniques, which are increasingly recognized as key components of next-generation cultural heritage monitoring systems [9,17,18,19,20,21,22].

## 5. Conclusions

This paper has presented the design, implementation, and experimental validation of a family of low-cost low-power LoRaWAN-based wireless sensing systems specifically developed for preventive conservation in CH environments. Rather than proposing an isolated sensing device, this work introduces a modular monitoring framework composed of complementary fixed and mobile sensing platforms capable of addressing different monitoring requirements encountered in historical buildings and archaeological sites.

The proposed systems include fixed wireless nodes for environmental monitoring, visitor counting, airflow analysis, crack monitoring, and volumetric water content measurements, together with mobile sensing platforms integrated into UAVs and quadruped robots for monitoring areas that are difficult or unsafe to access using conventional static installations. All sensing devices were designed following common principles of low power consumption, reduced physical footprint, modularity, low manufacturing cost, and native LoRaWAN connectivity, allowing long-term autonomous operation with minimal intervention on protected heritage structures.

A key contribution of this work is the integration of these heterogeneous sensing technologies within a common wireless monitoring architecture, enabling complementary monitoring strategies that combine continuous fixed measurements with flexible mobile inspections. Unlike many existing solutions that focus on a single sensing modality or a specific application, the proposed framework supports multiple environmental variables, visitor monitoring, and robotic inspection within the same technological framework.

The proposed monitoring systems were experimentally validated through real deployments in five pilot CH sites located in four European countries, including archaeological sites, historical buildings, castles, underground heritage, and religious monuments. These deployments demonstrate the scalability, adaptability, and robustness of the proposed architecture under diverse environmental and operational conditions.

From the perspective of preventive conservation, the proposed systems provide several practical benefits. Their non-invasive installation minimizes the impact on historical structures, while their low energy consumption and LoRaWAN communication enables long-term autonomous monitoring with limited maintenance requirements. Continuous acquisition of environmental and visitor-related information supports the early detection of potentially harmful conditions, allowing conservation managers to make informed decisions before significant deterioration occurs. Furthermore, the modular architecture facilitates future integration with digital twins, artificial intelligence algorithms, and predictive maintenance tools developed within the ARGUS project.

Overall, the results demonstrate that the proposed LoRaWAN-based monitoring approach constitutes a scalable, flexible, and cost-effective solution for long-term monitoring of CH assets. The combination of fixed and mobile sensing platforms provides a comprehensive approach that extends beyond traditional environmental monitoring and contributes to the development of next-generation smart preventive conservation systems.

## Figures and Tables

**Table 1:** Tailored systems developed for the different pilot sites.

System	Type	Deployment
Air flow monitoring	Fixed wireless node	Wine cellars of Baltanás
Visitor monitoring	Fixed wireless node	Wine cellars of Baltanás
Internal temperature and humidity (TH) monitoring	Fixed wireless node	Schenkenberg Castle, Wine cellars of Baltanás
Volumetric water content (VWC) and internal TH monitoring	Fixed wireless node	Ranverso Abbey, Schenkenberg Castle
Wind monitoring	Fixed wireless node	Ranverso Abbey, Archaeological site of Delos
Pollution monitoring	Fixed wireless node	Ranverso Abbey
Gas monitoring	UAV mobile platform	Schenkenberg Castle
Gas monitoring	Quadruped mobile platform	Wine cellars of Baltanás

**Table 2:** Pollution sensors selected for the prototype.

Sensor Name	Technology Used	Gas Measurement	Function
Sensirion SEN55	Optical scattering, MOX, temperature/humidity sensor	PM1.0/2.5/4/10, VOCs, NOx, temperature, humidity	Provides baseline data on air quality and microclimate; particles cause surface damage; VOCs/NOx can form acidic compounds; fluctuations in temperature and humidity lead to physical stress.
DFRobot Gravity SO_2_	Electrochemical	SO_2_	High-precision monitoring of corrosive acidic gases to prevent degradation through sulfation of materials such as stone and gypsum.
MiCS-6814	MOX	CO, NO_2_, NH_3_	Monitoring of traffic-related pollutants (CO, NO_2_) and agriculture/biological decomposition (NH_3_), with early warnings of material degradation risks.
Sensirion SCD41	NDIR	CO_2_	Measures occupancy levels and ventilation efficiency. In high-humidity environments, CO_2_ can form carbonates, accelerating the deterioration of cultural objects.

**Table 3:** Analysis of data collected by the V2 system.

Day	Day of the Week	Entry	Exit	Net Flux	Analysis
1	Friday	22	21	+1	On weekdays, traffic flows normally.
2	Saturday	2	1	+1	On weekends, there is practically no traffic.
3	Sunday	0	1	−1	On weekends, there is practically no traffic.
4	Monday	34	33	+1	On weekdays, traffic volume returns to normal.
5	Tuesday	34	29	+5	Weekdays, peak hours
6	Wednesday	31	31	0	During the week, a balance between incoming and outgoing traffic.
7	Thursday	26	23	+3	On weekdays, traffic flows normally.

**Table 4:** Summary of system validation results and performance indicators.

System	Validation Evidence	Main Outcome
Airflow and PIR monitoring	Deployment in underground cellars	Successful identification of airflow patterns associated with cellar ventilation conditions
Visitor monitoring V2	Controlled testing at ITEFI	Vehicle counting accuracy > 96% after excluding identified interference events
TH + VWC monitoring	Deployment in wall cracks	Continuous monitoring over approximately three months, capturing a seasonal temperature decrease from ~11 °C to ~3 °C together with gradual variations in volumetric water content inside the monitored wall.
MEMS anemometer	Comparison with commercial station	High temporal correlation for wind speed measurements (R = 0.904), demonstrating accurate tracking of wind speed dynamics and good agreement in overall wind direction trends.
Pollution monitoring	Multi-week deployment in Ranverso Abbey	Successful long-term monitoring of indoor air quality, detecting occupancy-related CO_2_ peaks (up to ~680 ppm) together with variations in VOCs and other pollutants.
Overall framework	Five pilot sites in four countries	Successful monitoring of more than 10 environmental and anthropogenic variables, demonstrating the adaptability and scalability of the proposed LoRaWAN-based monitoring framework.

## Data Availability

Data will be made available upon request.
